# The loss of B7-H4 expression in breast cancer cells escaping from T cell cytotoxicity contributes to epithelial-to-mesenchymal transition

**DOI:** 10.1186/s13058-023-01721-5

**Published:** 2023-10-04

**Authors:** Linlin Zhou, Jichun Wu, Mei Ruan, Yonglei Xiao, Hailin Lan, Qiongwen Wu, Chen-Wei Yu, Qiuyu Zhang

**Affiliations:** 1 Institute of Immunotherapy, Fujian Medical University, Fuzhou, China; 2 School of Basic Medical Sciences, Fujian Medical University, Fuzhou, China; 3 Department of Statistics and Information Science, Fu Jen Catholic University, New Taipei City, Taiwan

**Keywords:** B7-H4, Breast cancer, EMT, Stemness, T cell cytotoxicity

## Abstract

**Background:**

B7 homology 4 (B7-H4), a potential target for cancer therapy, has been demonstrated to inhibit T cell cytotoxicity in the early stages of breast cancer. However, B7-H4 manipulating breast tumor immune microenvironment (TIME) in the tumor progression remains unknown.

**Methods:**

We engineered T cells with B7-H4-specific chimeric antigen receptors (CARs) and performed a T cell co-culture assay to characterize B7-H4 expression level in breast cancer cells escaping from T cell cytotoxicity. We generated B7-H4 knockout (KO) and overexpression (OE) breast cancer cells to determine the epithelial-to-mesenchymal transition (EMT) and stemness characteristics in vitro and in vivo, including tumor proliferation, migration, metastasis and chemoresistance. The Cancer Genome Atlas breast cancer database was accessed to investigate the correlation between B7-H4 expression levels and EMT characteristics in patients with breast cancer.

**Results:**

Our result found that B7-H4 expression level was significantly reduced in a subset of breast cancer cells that escaped from the cytotoxicity of B7-H4 CAR-T cells. Compared with wild type cells, B7-H4 KO cells prompt EMT and stemness characteristics, including migration, invasion and metastasis, and OE cells vice versa. The increase in H3K27me3 in KO cells confirmed the epigenetic reprogramming of cancer stem cells. The IC50 of doxorubicin or oxaliplatin significantly increased in KO cells, which was in agreement with a decrease in OE cells. Moreover, a trend of downregulated B7-H4 from stage I to stage II breast cancer patients indicates that the low-expressing B7-H4 breast cancer cells escaping from TIME have spread to nearby breast lymph nodes in the cancer progression.

**Conclusions:**

Our study illuminates the novel role of renouncing B7-H4 in breast cancer cells through immune escape, which contributes to EMT processes and provides new insights for breast cancer treatments.

**Supplementary Information:**

The online version contains supplementary material available at 10.1186/s13058-023-01721-5.

## Background

Breast cancer is the most commonly diagnosed cancer and the leading cause of cancer death among women worldwide [1]. Breast cancer comprises different pathological subtypes and has infiltrating immune cells or therapeutic targets heterogeneously presented within and between patients [2]. The heterogeneity of breast tumor immune microenvironment (TIME) is considered the major cause of treatment failure in cancer immunotherapy. The use of anti-programmed death-ligand 1 (anti-PD-L1), programmed death-1 (anti-PD-1) and cytotoxic T-lymphocyte-associated protein 4 (anti-CTLA-4) has been shown to increase life expectancy in patients with certain cancers. However, these molecules are in fact not valuable for all cancers [3, 4]. The factors affecting immunotherapy mainly include PD-L1 expression levels, the content of tumor infiltrating T cells (TILs), tumor mutation burden, microsatellite instability, function of gene mismatch repair and the patient history of chemoradiotherapy [5, 6]. Previous evidence has revealed the clinical efficacy of PD-1/PD-L1 antagonists in a small group of metastatic breast cancer patients. It has been shown that breast cancer patients with triple negative, PD-L1 positive and higher levels of TILs will have better clinical outcomes [7]. Low immunogenicity and T cell infiltration with immunosuppressive tumor microenvironment impede the success of immunotherapy in breast cancer [8]. Ipilimumab (anti-CTLA-4 antibody) has shown promising results in treating melanoma, but progress has been slow in other cancer types because of low response rate and immunotherapy-related adverse events. [9, 10]. Therefore, it is critical to develop alternative immune therapeutic targets that might be functional in the non-responders.

Members of the B7 family of coregulatory molecules have been shown to play a crucial role in regulating tumor-specific response [11]. B7-H4, also known as V-set domain containing T cell activation inhibitor 1 (*VTCN1*/B7x/B7 homolog4/B7S1), is a type 1 transmembrane protein that belongs to co-inhibitory B7 family ligands, which has been reported to exert critical effects on the inhibition of T cell-mediated immune response. B7-H4 was found to be constitutively expressed in several cancer cells including ovarian cancer [12], prostate cancer [13], melanoma [14] and invasive ductal and lobular breast cancers [15–17]. Previous studies including ours have demonstrated that B7-H4 in breast tumor cells is a negative regulator of CD8 T cell activation, expansion and cytotoxicity, resulting in low T cell infiltration in TIME [15]. Conversely, tumor growth inhibition was not observed in the B7-H4^−/−^ mouse model, and the absence of B7-H4 leads to a reduction in CTLs (Cytotoxic T-Lymphocyte) granzyme B levels and the inability of tumor-specific T cells in the breast TIME [18]. These inconsistent findings might be due to the different tumor models and the heterogeneity of TIME.

Evidence shows that in the estrogen receptor-positive (ER+) breast cancer cell model, the transferring tumor cells result in lower metastasis, enhanced survival and decreased tumor infiltration of immunosuppressive cells in B7-H4 knockout compared to wildtype mice [19]. These results suggest that the expression of B7-H4 within the breast TIME by infiltrating immune cells is hypothesized to promote immune evasion. Epithelial-to-mesenchymal transition (EMT) is a typical embryonic development process and has long been linked to increasing invasiveness, favoring escape from the primary tumor site and thus metastasis [20]. In addition to immune invasion and chemoresistance, invasiveness and cancer cell stemness are the most critical properties of tumor development in EMT processes. Previous reports show that PD-L1 production by coordinating EMT processes modulates immunoresistance toward CTLs [21, 22]. These findings suggest that EMT processes, including invasiveness and stemness, might manipulate switching PD-L1 expression and govern immunotherapeutic responses in human breast cancer cells.

Most studies have focused on B7-H4 expression and its regulatory mechanism in immune cells. In the present study, we surprisingly found a dynamic downregulation of B7-H4 expression when tumor cells escaped from cytotoxicity of B7-H4 CAR-T cells, which is similar to the tendency of B7-H4 expression from stage I to stage II in human breast cancer patients. We further demonstrate that the absence of B7-H4 expression in breast cancer cells promotes EMT and stem cell differentiation, resulting in tumor migration, metastasis and chemoresistance. Our study presents new insight into the precise strategy of adoptive T cell therapy targeting breast cancer cells at different stages of tumor progression.

## Methods

### Human cells and cell culture

Human breast cancer cell lines were purchased from the American Type Culture Collection (ATCC), including SK-BR-3(SKBR3), MCF-7 (MCF7), MDA-MB-231, MDA-MB-436, MDA-MB-468, T-47D and BT-474. Tumor cells were cultured in DMEM or RPMI-1640 medium (Corning, NY, USA) with 10% fetal bovine serum (FBS), penicillin (100 U/mL), streptomycin (100 μg/mL) (Gibco, NY, USA), and cell lines were regularly tested for mycoplasma negative. All cells were incubated at 37 °C in a humidified chamber containing 5% CO_2_.

### The construction of tumor cell lines with B7-H4 gene knockout or overexpression

B7-H4 gene knockout cells (SKBR3-KO) were generated using the CRISPR/Cas9 system by infecting a custom-made lentivirus vector containing the Cas9 gene and sgRNA targeting the sequence of human B7-H4, and then, single cell clones were isolated by serial dilutions. B7-H4-overexpressing tumor cells (MDA-MB-231-OE and MCF7-OE) were prepared by transfection of the pcDNA-hB7-H4 plasmid encoding full-length human B7-H4 into MDA-MB-231 and MCF7 cells. G418 (Corning, NY, USA) was used in antibiotic selection to obtain stable clones.

### Immunoblotting

Immunoblotting was performed using standard techniques: Cell lysates were produced in RIPA lysis buffer (Abcam, MA, USA) supplemented with protease/phosphatase inhibitor (Cell Signaling Technology, USA). The protein concentration was quantified using a BCA protein assay kit (Beyotime Biotechnology, Shanghai, China). Lysates were separated by SDS-PAGE and then transferred onto PVDF membranes (Millipore, MA, USA). After blocking with the 5% milk blocking buffer, the membranes were incubated overnight at 4 °C with the primary antibodies: rabbit anti-human B7-H4 (clone D1M8I, #14572S) and anti-β-actin (clone13E5, #4970S). After incubated with secondary anti-rabbit antibodies conjugated to horseradish peroxidase (HRP) (1:10,000), the membranes were detected using enhanced chemiluminescent (ECL) HRP substrate (Vazyme, Nanjing, China). All the antibodies were purchased from Cell Signaling Technology (CST, MA, USA).

### Flow cytometry

Tumor cells were resuspended in 100 μl staining buffer after blocking Fc receptors, then incubated with APC-conjugated anti-B7-H4 (cloneMIH43, #358,108) or isotype control (BioLegend, CA, USA) for 30 min. After washing and centrifuging in staining buffer, cells were fixed and permeabilized, and then performed intracellular staining of PE-conjugated anti-Ki67 (BioLegend, CA, USA). After staining, cells were evaluated using BD FACS Verse and analyzed using FlowJo 10 (BD Biosciences, CA, USA).

### Proliferation assay and cell cycle analysis

SKBR3-WT and -KO cells were plated in 96-well plates at 1000 cells/well. Cell growth rates were monitored using a Cell Counter (BioRad, CA, USA) for 7 days. For cell cycle analysis, cells were grown to 70–80% confluency, treated with RNase A, stained with propidium iodide (Sigma-Aldrich, MO, USA) and then subjected to flow cytometry with BD FACS Verse, and cell cycle distribution was calculated using the FlowJo 10. Immunoblotting was used to analyze the expression levels of cell cycle regulators in SKBR3-KO and SKBR3-WT. Rabbit anti-human primary antibodies were obtained from Cell Signaling Technology (CST, MA, USA), including anti-Cyclin D1(clone 92G2, #2978), anti-Cyclin E2(#4132), anti-CDK2(clone 78B2, #2546), anti-CDK4(clone D9G3E, #12,790), anti-p27(clone D69C12, #3686) and anti-GAPDH (clone D16H11, #5174) antibodies.

### Preparation of B7-H4 CAR-T cells

High titers of the lentivirus vectors pMSCV-H4-CAR were generated by transient transfection and used to transduce HEK 293 T cells at MOI of 5. Peripheral blood mononuclear cells (PBMCs) were isolated by density gradient centrifugation over Ficoll-Paque (GE Healthcare, Little Chalfont, UK) and activated with anti-CD3/CD28 Dynabeads (Thermo Fisher Scientific, MA, USA) combined with 50 U/mL recombinant human IL-2 (Peprotech, NJ, USA) for 24 h, and then, PBMCs were cultured with the desired lentivirus at appropriate MOI for 24 h. The purity and phenotype of CAR-T cells were verified by flow cytometry.

### T cell cytotoxicity assay

To analyze B7-H4 CAR-T cell cytotoxicity, 5 × 10^4^ SKBR3 (WT and KO) cells were labeled with 3 μM carboxyfluorescein diacetate succinimidyl ester (CFSE, Thermo Fisher Scientific, MA, USA) as target cells and then incubated with B7-H4 CAR-T cells 24 h at various effector-to-target ratios. Killing effect was evaluated by a cell death marker (LIVE/DEAD^®^ Fixable Dead Cell Stain Kits, Thermo Fisher Scientific, MA, USA) using flow cytometry. Live cell imaging and data analysis were performed using EVOS FL Auto microscope (Thermo Fisher Scientific, MA, USA).

### Modified transwell migration assay induced by T cell cytotoxicity

The modified transwell migration assay was used to identify and characterize tumor cell escape from T cell cytotoxicity by migration. SKBR3 cells (5 × 10^4^) were labeled with 3 μM CFSE as target cells and then incubated with B7-H4 CAR-T cells or activated T cells for 24 h in the upper chamber of an 8.0-μm transwell (Corning, NY, USA). Cell culture media containing 10% FBS was placed in the bottom well of the lower chamber in a 24-well plate. We took photographs of 5 fields randomly and counted the number of CFSE labeled tumor cells migrated to the lower chamber, and then, we collected cells and analyzed the all samples by flow cytometry. Tumor cells migrated from the upper to the lower were collected and analyzed B7-H4 expression.

### Wound healing assay

Cells were seeded in 24-well plates and cultured in a complete medium overnight. When the confluence reached 90%, cells were scraped across the well gently with a micropipette tip, and floating cells were washed away with PBS. Cell migration was monitored at 0 h, 24 h and 48 h using a microscope, and distance between the two edges of the wound was calculated.

### Transwell invasion assay

2 × 10^4^ cells were seeded in the upper side chamber of an 8.0-μm transwell in a 24-well culture plate (Corning, NY, USA) with the upper membrane covered by Matrigel (BD Biosciences, CA, USA). The lower chamber comprised DMEM containing 10% FBS and then we took photographs of 5 fields randomly and counted the number of cells passing through the matrigel-coated membrane after the samples were fixed and stained with 0.4% trypan blue solution (Thermo Fisher Scientific, MA, USA) to evaluate the invasiveness of cells on the membrane.

### Quantitative RT-PCR

Total RNA isolation from cells was performed using RNA Isolating Kit (Vazyme, Nanjing, China), and cDNA was synthesized using PrimeScript RT Master Mix (TaKaRa, Tokyo, Japan) following the manufacturer’s protocol. The following qPCR procedure was initiated by 3 min at 95 °C, and followed by 40 cycles of 15 s at 95 °C, 30 s at 55 °C, and 30 s at 72 °C. The expression levels of the target genes were calibrated by the internal control. The C_T_ calculated quantification of all the samples by the software and relative fold changes were calculated using the 2^−ΔΔCT^.

### RNA-Seq

Total RNA was extracted using TRIzol (Thermo Fisher Scientific, MA, USA) reagent. Expression values of EMT and stemness-associated genes and transcription factors were evaluated from the transcriptome RNA sequencing data of SKBR3 (WT and KO), MDA-MB-231(WT and OE) and MCF7 (WT and OE) cell samples. The unit used to establish the fold change in RNA-seq analysis is TPM. We compared the TPM value of each gene’s expression between two cell lines (KO and WT, or WT and OE) in the RNA-seq analysis. Then, the ratio of gene expression levels between the two samples was calculated to establish the fold change. Log2 scale is represented as the logarithm base 2 of the fold change.

### Mouse xenograft model

8-week-old adult male BABL/c nude mice were purchased from Beijing Vital River (Beijing, China) and housed in pathogen-free facilities in the Experimental Animal Centre of Fujian Medical University. SKBR3 (WT and KO), MDA-MB-231 (WT and OE) and MCF7 (WT and OE) cells were embedded in matrigel (Corning, NY, USA) and then subcutaneously injected into the right flank at limiting dilutions. Tumor length and width were measured with a caliper every other day to calculate tumor diameter using the equation (length + width)/2. Mice were killed when tumors diameter reached the maximum allowed size (15 mm) or when signs of ulceration were evident. For the lung metastasis tumor model, the mice were randomized in groups, and tumor cells were resuspended in 100 μl of PBS and injected into the tail vein to generate metastasis in nude mice. The mice were euthanized 4 weeks after the injection of cancer cells, and lungs were collected. All procedures performed in studies involving animals were approved by the Fujian Medical University Institutional Animal Care and Use Committee (IACUC) in accordance with the ethical standards.

### HE and IHC staining

HE staining of lung tissue was conducted to evaluate the metastasis of cancer cells within the lung. The lung tissues were fixed with 4% paraformaldehyde overnight, then embedded in paraffin and sliced into 8 μm thick sections. After dewaxed with xylene, the dehydrated with increasing ethanol concentration, slides were stained with HE solution. For Immunohistochemistry (IHC) staining, human breast cancer tissues were fixed and paraffin-embedded according to the manufacturer’s instructions. The sections were immunolabeled with mouse anti-B7-H4 antibodies (clone 6H3, in house), followed by anti-mouse peroxidase kit (Vector, CA, USA) labeling, respectively, according to the manufacturer’s instructions. The slides were photographed under an optical microscope. Expression levels for B7-H4 were scored semiquantitatively based on staining intensity (SI) and percentage of positive cells (PP). An immunoreactive score (IRS) was calculated using the following formula: IRS = PP × SI [23]. All procedures performed in studies involving human tissues were in accordance with the ethical standards of the institutional and/or national research committee and with the 1964 Helsinki declaration and its later amendments or comparable ethical standards.

### Immunofluorescence

Lung tissue sections or cells growing on the glass slide (20,000 cells/slide) were fixed with 4% paraformaldehyde, permeabilized by 0.1% Triton and stained with the indicated concentrations of rabbit anti-human primary antibodies, including anti-E-cad (clone 24E10, #3195), anti-N-cad (clone D4R1H, #13116 T), anti-H3K27me3 (clone C36B11, #9733 T), and anti-H3K9me3 (clone D4W1U, #13969 T), followed by Alexa Fluor-488-conjugated donkey anti-rabbit secondary antibody (1:1000). All the antibodies were obtained from Cell Signaling Technology (CST, MA, USA). The antibody-labeled cells on coverslips were stained with DAPI (1:1000, Sigma-Aldrich, MO, USA) for 2 min. Cells were observed under an inverted microscope using 405 and 488 nm lasers to visualize nuclei and target protein expression, respectively. Then, we randomly selected photographs of 4 fields for fluorescence quantitative analysis to evaluate the relative optical density by Image J.

### MTT assay for cytotoxicity

Breast cancer cells were seeded into 96-well plates (10,000 cells/well) and cultured in complete medium for 24 h and then treated with different concentrations (0–500 μM) of chemotherapy agents, including Doxorubicin (Dox) (MCE, NJ, USA), Gefitinib (Gef) (Sigma-Aldrich, MO, USA), Oxaliplatin (Oxa) (Sigma-Aldrich, MO, USA) and Fluorouracil (5-FU) (Sigma-Aldrich, MO, USA). After another 20 h incubation, 10 μl MTT was added to each well for 4 h at 37 °C. The formazan produced by viable cells was dissolved with DMSO (Sigma-Aldrich, MO, USA) and then measured the absorbance at 570 nm.

### Sphere formation assay

500 cells were seeded in 96-well ultra-low attachment plates (Corning, NY, USA) in a serum-free tumorsphere culturing medium containing epidermal growth factor (20 ng/ml), basic fibroblast growth factor (20 ng/ml) and B27 supplements. Tumor sphere formation was monitored using a microscope. Total RNA was isolated from the spheres on the 14th day for gene expression analysis by real-time PCR.

### Acquisition of gene expression profiles from TCGA datasets

The RNA-seq data and clinical information of breast cancer samples (TCGA BRCA) were collected and downloaded through UCSC Xena and TGCA real-time hub. The gene expression analysis in normal breast tissues was collected using the Genome Tissue Expression (GTEx) portal.

### GSEA (gene set enrichment analysis)

GSEA was performed on various gene signatures by comparing gene sets from the Molecular Signature Database (MSigDB) database or published gene signatures. Gene sets with a false discovery rate (FDR) value < 0.25, *p* value < 0.05, and ∣normalized enrichment score (NES) ∣ ≥ 1 were considered statistically significant [24, 25].

### Statistical analysis

All experiments were performed three or more times independently under similar conditions. Results are shown as the means, and the error bars represent the standard error of the mean (SEM), unless stated otherwise. GraphPad Prism 9 was used to generate graphs and perform statistical analysis. P values were calculated by unpaired two-tailed Student’s t test, one-way ANOVA and chi-squared test. Statistically significant results are represented as follows: *p < 0.05, **p < 0.01, ***p < 0.001 and ****p < 0.0001.

## Results

### The absence of B7-H4 is crucial for breast cancer cell immune escape

**Fig. 1:**
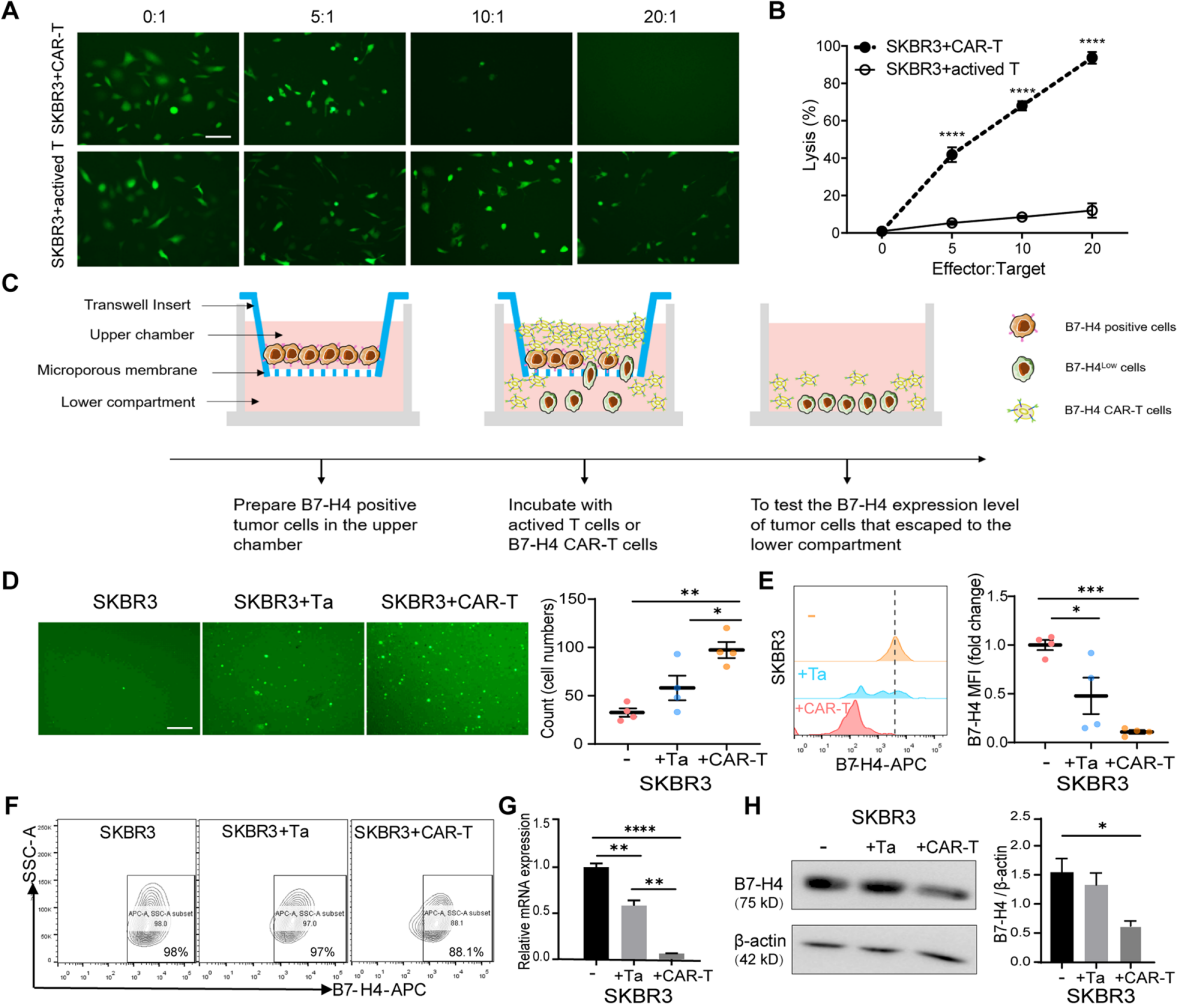
The absence of B7-H4 is essential for tumor cells to escape from cytotoxic T cells. **A, B** The cytotoxic effect of B7-H4 CAR-T cells and non-specifically activated T cells on CFSE labeled SKBR3 cells at the indicated effector/target (E/T) ratios. The residual living SKBR3 cells were imaged by microscopy (**A**) and the cytolytic percentages were calculated in the killing assay (**B**). Scale bar = 50 μm. **C** A schematic co-cultured transwell model presents simulating T cell-mediated cytotoxicity in TIME. **D** T cell-induced escape of CFSE-labeled SKBR3 cells into the lower chamber was observed by microscopy (left graph) and quantified by flow cytometry analysis (right graph). Scale bar = 100 μm. **E** B7-H4 expression levels of SKBR3 cells in the lower chamber of different groups were quantified by flow cytometry analysis. **F–H** B7-H4 expression levels of residual living SKBR3 cells were analyzed by flow cytometry (**F**), qPCR (**G**) and western blot (**H**) after co-culture with non-specific activated T cells and B7-H4 CAR-T cells, respectively. The data represent the mean ± SEM from three independent experiments, and statistical significance was determined by two-tailed unpaired *t* test (**B**) or one-way ANOVA (**D**–**H**). Ta: activated T cells. (*p < 0.05, **p < 0.01, ***p < 0.001, ****p < 0.0001)

Our previous study demonstrated that B7-H4 was highly expressed in breast invasive ductal carcinomas, and B7-H4 overexpression prevented the cytotoxicity of antigen specific CD8 T cells against mouse tumor cells [15]. In this study, we analyzed different breast cancer cell lines and identified two types of cell lines with the constitutive surface expression of B7-H4, including SKBR3 (HER2-enriched breast cancer cells) and MDA-MB-468 (triple-negative breast cancer cells, TNBC) (Additional file 1: Fig. S1A). To evaluate the potential impact of human B7-H4 on antitumor immunity, we engineered T cells with B7-H4-specific chimeric antigen receptors (CARs) and performed a cytolytic T cell assay (Additional file 1: Fig. S1B). A decrease in cancer cell viability was observed at effector/target (E/T) ratios from 5:1 to 20:1 when B7-H4 CAR-T cells were co-cultured with SKBR3 cells compared with non-specifically activated T cells (Fig. 1A), suggesting that B7-H4 CAR-T cells exhibited cytotoxic characteristics. Our findings indicated that both specific B7-H4 CAR-T cells and non-specifically activated T cells are capable of killing B7-H4 positive cells, and the highest cytolytic cancer cells were most significantly seen in co-cultured B7-H4 CAR-T cells when compared to non-specifically activated T cells (Fig. 1B). To further reveal the potential mechanism of tumor cells escaping CAR-T cell-mediated cytotoxicity in tumor microenvironment, we developed a modified transwell assay to quantify the migration of B7-H4 positive tumor cells (Fig. 1C). Notably, the highest numbers of SKBR3 cells were observed in the lower chamber when they co-cultured with B7-H4 CAR-T cells compared to that with activated T cells, suggesting that more breast cancer cells were escaped from the cytotoxicity of B7-H4 CAR-T cells (Fig. 1D). More importantly, the expression levels of B7-H4 on these escaping breast cancer cells were significantly reduced after co-culture with B7-H4 CAR-T cells, and a slight decrease in B7-H4 expression was seen on SKBR3 escaping cells co-culture with activated T cells (Fig. 1E). However, no change of B7-H4 expression was observed on SKBR3 escaping cells co-culture without B7-H4 CAR-T cells demonstrating by FACS (Fig. 1F), qPCR (Fig. 1G) and immunoblotting (Fig. 1H). Similar results were obtained from the escaping cells when MDA-MB-468 cells co-cultured with B7-H4 CAR-T cells (Additional file 1: Fig. S1C–G). These findings indicated that B7-H4 expression in breast cancer cells might be variated in modulating the immune microenvironment.

### B7-H4 deficiency promotes human breast cancer cell proliferation and migration

**Fig. 2:**
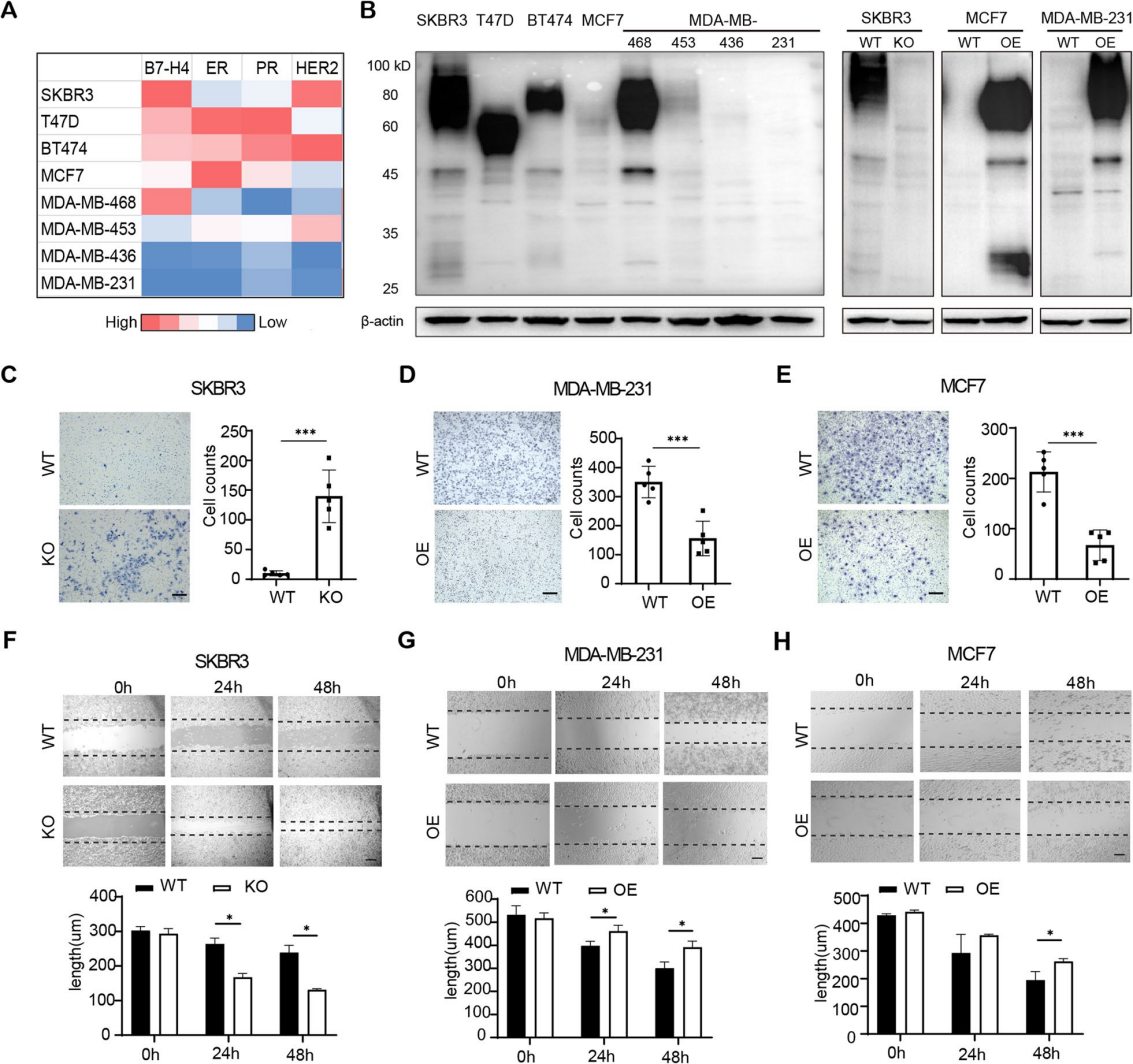
B7-H4 interferes human breast cancer cell proliferation and migration. **A** The mRNA expression levels of B7-H4, hormone receptors (ER and PR) and HER2 in different types of breast cancer cell lines were analyzed using CCLE database. **B** The protein expression levels of B7-H4 were analyzed by immunoblotting in various human breast cancer cell lines (left graph) and the engineering breast cancer cells with B7-H4 knockout or B7-H4 overexpression (right graph). **C–E** Cell invasion ability was compared between wide type and engineering tumor cell lines including SKBR3, MDA-MB-231 and MCF7, using transwell matrigel invasion assay. **F–H** A wound healing assay detected cell migration analysis in paired tumor cell lines. Scale bar = 100 μm. Representative images and quantified data were shown in the graphs. Data are represented as mean ± SEM of three independent experiments, and statistical significance was determined by a two-tailed unpaired *t* test (*p < 0.05, ***p < 0.001). CCLE: Cancer Cell Line Encyclopedia; WT: wide type cell lines; KO: B7-H4 knockout cell lines; OE: B7-H4 overexpression cell lines

Previous studies suggest that B7-H4 expression is restricted to some human breast cancer cells in terms of the hormone receptor phenotypes [15, 26]. We expanded these studies by analyzing the expression of B7-H4 in a panel of human breast cancer cells using the Cancer Cell Line Encyclopedia (CCLE) database. In contrast to the previous reports, the results reveal abundant B7-H4 is detected in human breast cancer cell lines, including SKBR3, T-47D (HR+), BT-474 (HR+ and HER2-enriched) and MDA-MB-468 cells. Notably, B7-H4 expression was weakly or not detected in MCF7 (HR+), MDA-MB-453 (basal-like), MDA-MB-436 (TNBC) and MDA-MB-231 (TNBC) cells (Fig. 2A). The similar results are confirmed by immunoblotting (Fig. 2B, left graph). These results indicate that B7-H4 is heterogeneously expressed in different types of human breast tumor cell lines. Our results showed that B7-H4 expression levels in survival tumor cells were damped after treatment with B7-H4 CAR-T cells. To deeply understand the role of B7-H4 in human breast cancer cells, we herein generated B7-H4 knockout cells (SKBR3-KO) and B7-H4 overexpression cells (MCF7-OE and MDA-MB-231-OE) (Fig. 2B, right graph). Notably, cell proliferation was dramatically increased in SKBR3-KO when compared to wide type cells (SKBR3-WT) measured by Ki67 staining and trypan blue cell counting (Additional file 2: Fig. S2A, B). Correspondingly, the number of SKBR3-KO cells at the G0/G1 phase was decreased with subsequently increased in S phases when compared to SKBR3-WT cells (Additional file 2: Fig. S2C). In addition, we observed an increase in the expression of positive cell cycle regulators CDK2, CDK4, Cyclin D1 and E2, and a decline in the negative cell cycle regulator p27, in SKBR3-KO cells when compared with SKBR3-WT (Additional file 2: Fig. S2D). Thus, B7-H4 deficiency prompts cell proliferation and cell cycle exoneration in breast cancer cells. To determine whether B7-H4 has an effect on human breast cancer cell motility, a critical process during metastasis, we initially assessed the effect of B7-H4 on chemotactic and random migration using transwell assay and the cellular wound assay, respectively. These experiments were performed using growth-arrested cells to ensure that the effect of B7-H4 on cell proliferation would not be a confounding factor. As expected, cell migration was significantly increased in SKBR3-KO, MDA-MB-231-WT and MCF-7-WT cells, in comparison with SKBR3-WT, MDA-MB-231-OE and MCF7-OE cells (Fig. 2C–H). These findings demonstrate that B7-H4 reduces both proliferation and migration in human breast cancer cells.

### B7-H4 deficiency regulates EMT and stemness characteristics of human breast cancer cells

**Fig. 3:**
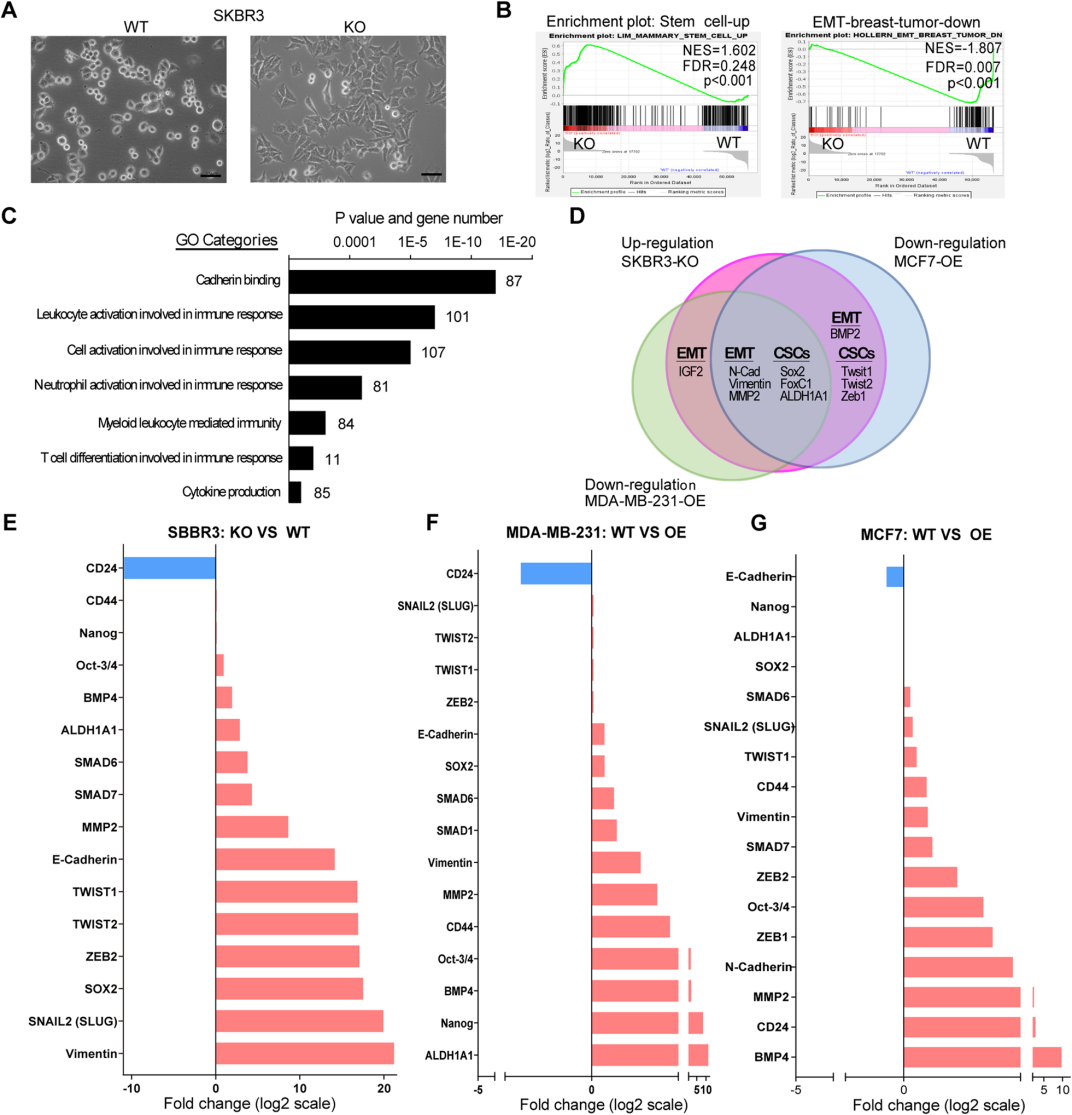
B7-H4 regulates breast cancer cell EMT and stemness genes. **A** Cell morphology was investigated in SKBR3-KO and -WT cells. Scale bar = 50 μm. **B** The identification of over- and under-represented gene groups and enriched pathways based on differentially expressed genes in SKBR3-KO and SKBR3-WT cells was performed by the GSEA analysis. **C** The differentially expressed genes of enriched biological functions compared to WT and SKBR3-KO cells were analyzed by Gene Ontology (GO). **D** Venn diagram shows the overlapping genes associated with EMT and cell stemness in SKBR3-KO, MCF7-OE and MDA-MB-231-OE cell lines. **E–G** RNA-seq results show the mRNA expression levels of EMT, cell stemness and self-renewal genes significantly altered in (**E**) SKBR3-KO, (**F**) MDA-MB-231-OE and (**G**) MCF7-OE cells versus respective control cells (Log 2 scale). The unit used to establish the fold change in RNA-seq analysis is TPM

After the surprising finding that B7-H4 negatively regulates breast cancer cell proliferation and migration, we assume that B7-H4 might regulate EMT. EMT is a potential mechanism by which tumor cells gain metastatic features such as losing cell–cell contacts and gaining the fibroblast-like morphology [20]. Moreover, through EMT, tumor cells acquire cancer stem cell (CSC) and chemoresistance properties [27]. Our results confirm that the fibroblast-like mesenchymal phenotypes were observed in SKBR3-KO cells compared to the SKBR3-WT cells (Fig. 3A). We have performed in vitro migration and invasion assays using SKBR3-WT and SKBR3-KO cells. As shown in Fig. 2, the migration and invasion ability of SKBR3-KO is much higher than that of SKBR3-WT cells. Therefore, there is a clear difference between the two cell lines in the metastatic potential. To further elucidate the role of B7-H4 in regulating breast cancer cell EMT, we initially conducted a comprehensive RNA-seq in three pairs of breast cancer cells with different B7-H4 expression levels. The differentially expressed genes of pairwise comparisons and the Gene Ontology (GO) analysis of the 556 expressed gene clusters revealed that SKBR3-KO cells when compared with SKBR3-WT cells, distributed some enrichment in regard to cadherin binding, cell activation involved in immune response and T cell differentiation involved in immune response (Fig. 3C). The Gene Set Enrichment Analysis (GSEA) further confirmed B7-H4 regulating several critical pathways, including stem cells and breast cancer proliferation (Fig. 3B). In addition, we found that EMT and breast cancer stem cell transcriptional signatures were upregulated in SKBR3-KO cells and downregulated in MCF7-OE or MDA-MB-231-OE cells (Fig. 3D). The upregulation of EMT markers including vimentin (*VIM*), *MMP2*, N-Cadherin (*CDH2*), *IGF-2*, *BMP2*, *SMAD6* and *SMAD7*, and potential CSC makers including *TWIST1*, *TWIST2*, *ZEB1*, *ZEB2*, SNAIL3 (*SNAI3*), *FOXC1* and *FOXC2* were observed in SKBR3-KO cells when compared with SKBR3-WT cells (Fig. 3E). In contrast, the downregulation of several EMT and potential CSC makers was observed in MDA-MB-231-OE cells (Fig. 3F) and MCF7-OE (Fig. 3G). To further confirm these results, we examined the mRNA and protein levels of EMT- and CSC-related genes using qPCR (Additional file 3: Fig. S3A–C) and immunoblotting (Additional file 3: Fig. S3D), with similar results being observed after comparison with the RNA-seq analysis. To demonstrate whether B7-H4 may mediate breast cancer stem cell differentiation, three-dimensional in vitro culture of single SKBR3 sphere-forming cells was used to differentiate the stemness (Additional file 3: Fig. S3E). Our results show that B7-H4 expression is slightly altered and stem cell markers including *ALDH1A1*, *ZEB1*, *SNAI3*, *TWIST1* and *CDH2* are increased compared to the two-dimensional cultures (Additional file 3: Fig. S3F). Our results suggest that B7-H4 plays an essential role in legitimizing breast cancer cells undergoing EMT processes and stemness.

### B7-H4 deficiency promotes breast cancer stem cell differentiation and chemoresistance

**Fig. 4:**
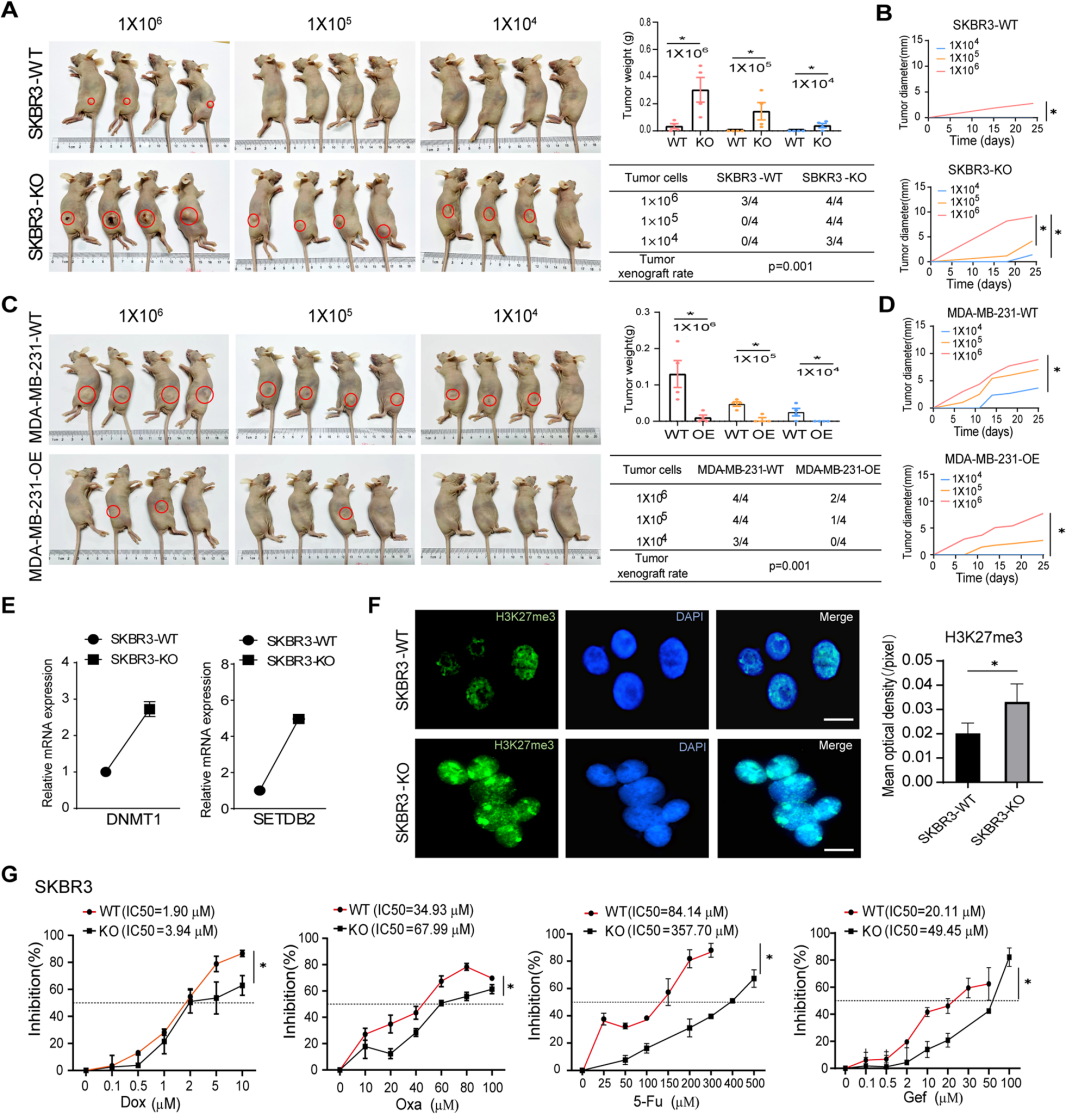
B7-H4 deficiency enhances breast cancer stem cells and chemoresistance. **A–D** In vivo tumorigenicity of breast cancer cells was performed in the subcutaneous xenograft mouse model at limiting dilutions. Tumors were isolated from nude mice 25 days postinoculation, and then, tumor mass and tumor xenograft rate were calculated (**A**, **C**). Tumor diameters in SKBR3 (**B**) and MDA-MB-231 (**D**) with or without B7-H4 KO and OE were measured every other day after xenografting. **E** The methylation markers *DNMT1* and *SETDB2* were examined by qPCR. **F** Immunofluorescence investigated the expression levels of H3K27me3 in SKBR3-WT and SKBR3-KO cells. Scale bar = 10 μm. **G** Quantifying IC50 values of commonly used chemotherapeutic agents on SKBR3-WT and SKBR3-KO cells were investigated by using MTT assay. Dox: Doxorubicin; Oxa: Oxaliplatin; 5-Fu: Fluorouracil; and Gef: Gefitinib. Data are represented as mean ± SEM of three independent experiments, and tumor xenograft rate was calculated by chi-squared test. Statistical assessment of the tumor growth curves was determined by one-way ANOVA (**B** and **D**). (*p < 0.05)

To demonstrate whether B7-H4 deficiency leads to breast cancer stem cell differentiation in vivo, SKBR3-KO, MCF7-OE and MDA-MB-231-OE cells were employed subcutaneous xenografting in nude mice at a limiting dilution to examine tumorigenicity, which is the gold standard assay that fulfills the criterion of CSCs. No tumors were seen after injection of 1 × 10^4^ and 1 × 10^5^ of SKBR3-WT cells. However, a significant tumor formation was observed after injection of 1 × 10^4^ and 1 × 10^5^ SKBR3-KO cells (Fig. 4A). Consistently, no tumors were seen after injection of 1 × 10^4^ of MDA-MB-231-OE cells and MCF7-OE cells, and tumor diameter was impaired in breast cancer cells with B7-H4 overexpression (Fig. 4C, D, Additional file 4: Fig. S4A, B). The tumor growth was significantly increased in the SKBR3-KO (Fig. 4A, B), MDA-MB-231-WT (Fig. 4C, D) groups when compared with the control groups respectively.

Previous reports indicate not only EMT contributes to chemoresistance but also CSCs are dynamic populations, which is critical for metastasis and chemoresistance [28, 29]. Our data show that the methylation makers *DNMT1* and *SETDB2* were increased in SKBR3-KO cells as compared with SKBR3-WT cells (Fig. 4E). The results suggest that B7-H4 deficiency leads to epigenetic reprogramming in human breast cancer cells and might promote the breast cancer stem cell differentiation. Thus, we investigated the expression of histone 3, lysine 9 (H3K9) and a histone 3, lysine 27 (H3K27) trimethylation which have been respectively associated with gene repression. The high expression levels of H3K27me3 (Fig. 4F) but not H3K9me3 (Additional file 4: Fig. S4C) were observed in SKBR3-KO cells as compared with SKBR3-WT cells, while H3K27me3 expression levels were inhibited in MDA-MB-231-OE cells (Additional file 4: Fig. S4D). Moreover, Gene Set Enrichment Analysis (GSEA) of RNA-seq revealed that genes related to the upregulation of H3K27me3 were enriched in the B7-H4 low expression group (SKBR3-KO and MDA-MB-231-WT cells) (Additional file 4: Fig. S4E), which indicates that B7-H4 deficiency indeed causes epigenetic reprogramming by modulating histone methylation. We confirmed the difference in H3K27me3 levels between SKBR3-WT and SKBR3-KO, and MDA-MB-231 WT and OE cells, respectively, by western blot analysis (Additional file 4: Fig. S4F). The results consistently demonstrated that loss of B7-H4 expression in breast cancer cells upregulates the level of H3K27me3. Corresponding to the upregulation of H3K27me3 in the B7-H4-deficient cell line, we observed that EZH2 (enhancer of zeste homolog 2), the core methyltransferase for H3K27me3, was elevated in SKBR3-KO cells and decreased in MDA-MB-231-OE cells (Additional file 4: Fig. S4G). These results support a potential link between B7-H4 dysregulation and histone methylation-mediated epigenetic reprogramming.

Chemoresistance is a typical phenotype in the acquisition of malignancy in breast tumor and is directly associated with the stemness of breast cancer cells. For further mechanistic insights into the role of B7-H4 in breast cancer cell chemoresistance, the chemosensitivity of Doxorubicin [30], Oxaliplatin [31, 32], Fluorouracil [33] and Gefitinib [34] was investigated in B7-H4-KO and B7-H4-OE cells by evaluating the IC50 values. These compounds are widely used therapies in treating breast cancer patients. However, resistance to these chemicals is also widespread in breast cancer patients, representing a significant challenge in clinical practice. Our results show that the IC50 for Doxorubicin (3.94 μM), Oxaliplatin (67.99 μM), Fluorouracil (357.70 μM) and Gefitinib (49.45 μM) was observed in the SKBR3-KO cells, which are much higher than WT cells. These findings are in agreement with a decrease in MDA-MB-231-OE cells (Additional file 4: Fig. S4H). Interesting, the significantly difference of IC50 was not seen in MCF7-OE cells (data not shown). Our results suggest that B7-H4 might at least in part mediate human breast cancer cell stemness and chemoresistance.

### B7-H4 deficiency promotes breast cancer cell metastasis

**Fig. 5:**
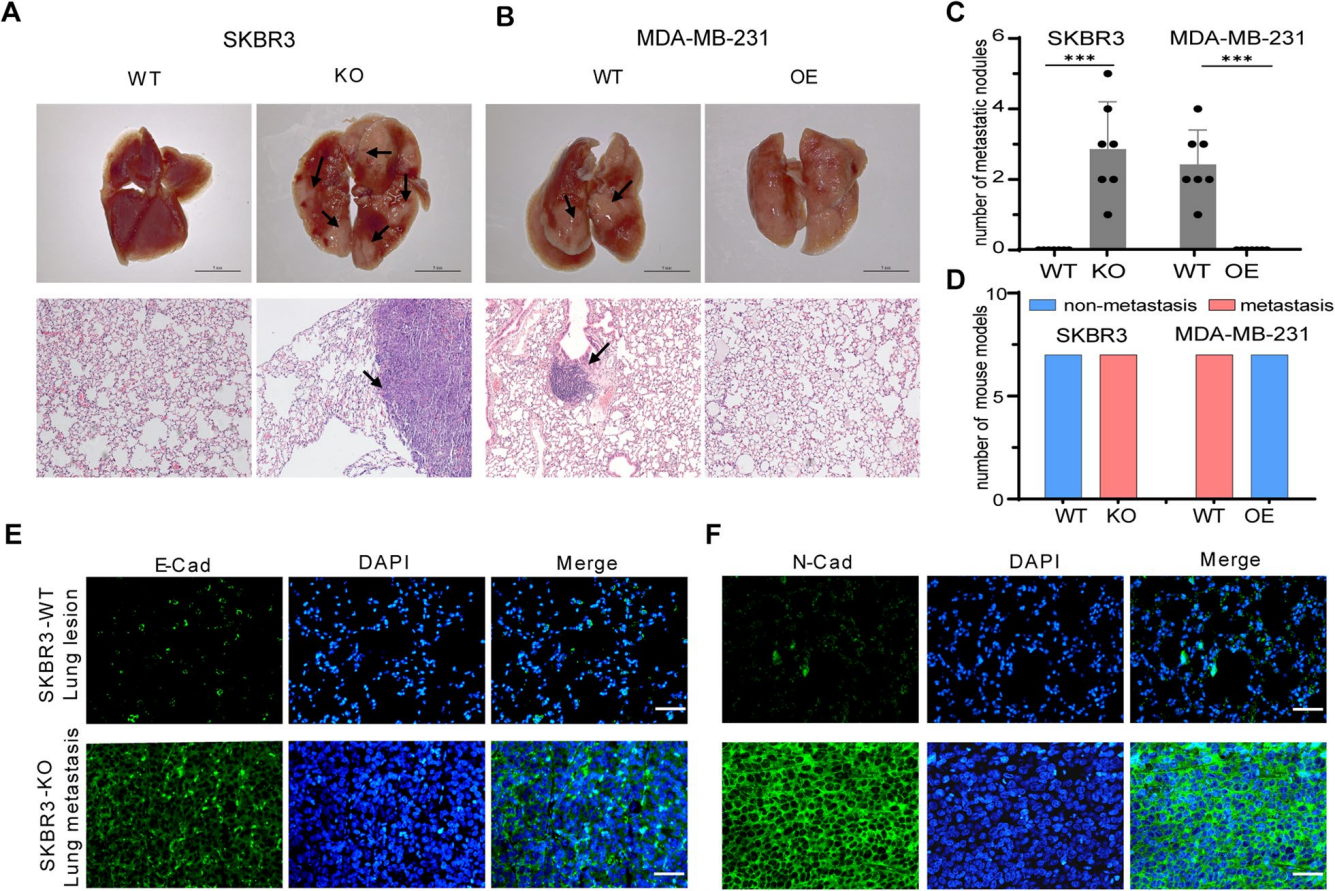
B7-H4 deficiency promotes breast cancer cell metastasis in vivo. **A, B** A commonly employed murine model to study breast cancer lung metastasis entails the injection of cancer cells via the tail vein to implant cells in the lung, and lung metastasis tissues were isolated 4 weeks after implantation. Scale bar = 5 mm. The representative lung tissue images and HE staining (Scale bar = 100 μm) of **A** SKBR3 (WT and KO) and **B** MDA-MB-231 (WT and OE) were shown. The black arrows represent the location of the metastatic tumors. **C** The number of metastatic nodules in representative lung tissue of each group was calculated. **D** The total number of mice with and without metastatic lung tumors (metastasis and non-metastasis) in each group (*n *= 5) was calculated. **E, F** The expression levels of (**E**) E-Cad and (**F**) N-Cad in SKBR3-WT and SKBR3-KO inoculated mouse lung tissues were investigated by immunofluorescence. Scale bar = 100 μm. E-Cad: E-Cadherin; N-Cad: N-Cadherin. Data are represented as mean ± SEM of three independent experiments, and statistical significance was determined by two-tailed unpaired *t* test. (***p < 0.001)

To determine the biological impact of B7-H4 signaling on breast cancer cells in vivo, we examined the tumorigenicity and lung metastasis in the immune-deficient mice model. SKBR3-KO, MCF7-OE and MDA-MB-231-OE cells were injected via the tail vein to implant cells in the lung. Strikingly mice injected with SKBR3-KO cells displayed prominent lung metastatic lesions compared to SKBR3-WT cells (Fig. 5A and Additional file 5: Fig. S5D). Lung metastatic lesions were also found in mice injected with MDA-MB-231-WT, whereas no metastases were observed in mice injected with MCF7, MCF7-OE and MDA-MB-231-OE cells (Fig. 5B and Additional file 5: Fig. S5). Histological quantification analyses further confirmed the breast cancer cell metastases in the lung (Fig. 5C, D). To further demonstrate B7-H4 deficiency promotes breast cancer cells metastasis via undergoing EMT, the expressions of E-Cadherin (E-Cad) and N-Cadherin (N-Cad) were examined by immunohistofluorescence. Our results confirmed that the lung metastasis tumor tissues were highly expressed N-Cad and/or E-Cad in SKBR3-KO injected mice (Fig. 5E, F). Our results suggest that the significant impact of B7-H4 signaling of breast cancer cells on metastasis is through legitimizing EMT transition.

### The fluctuation of B7-H4 expression levels among breast tumor stages might be associated with the immune escape of breast cancer cells in certain stages

**Fig. 6:**
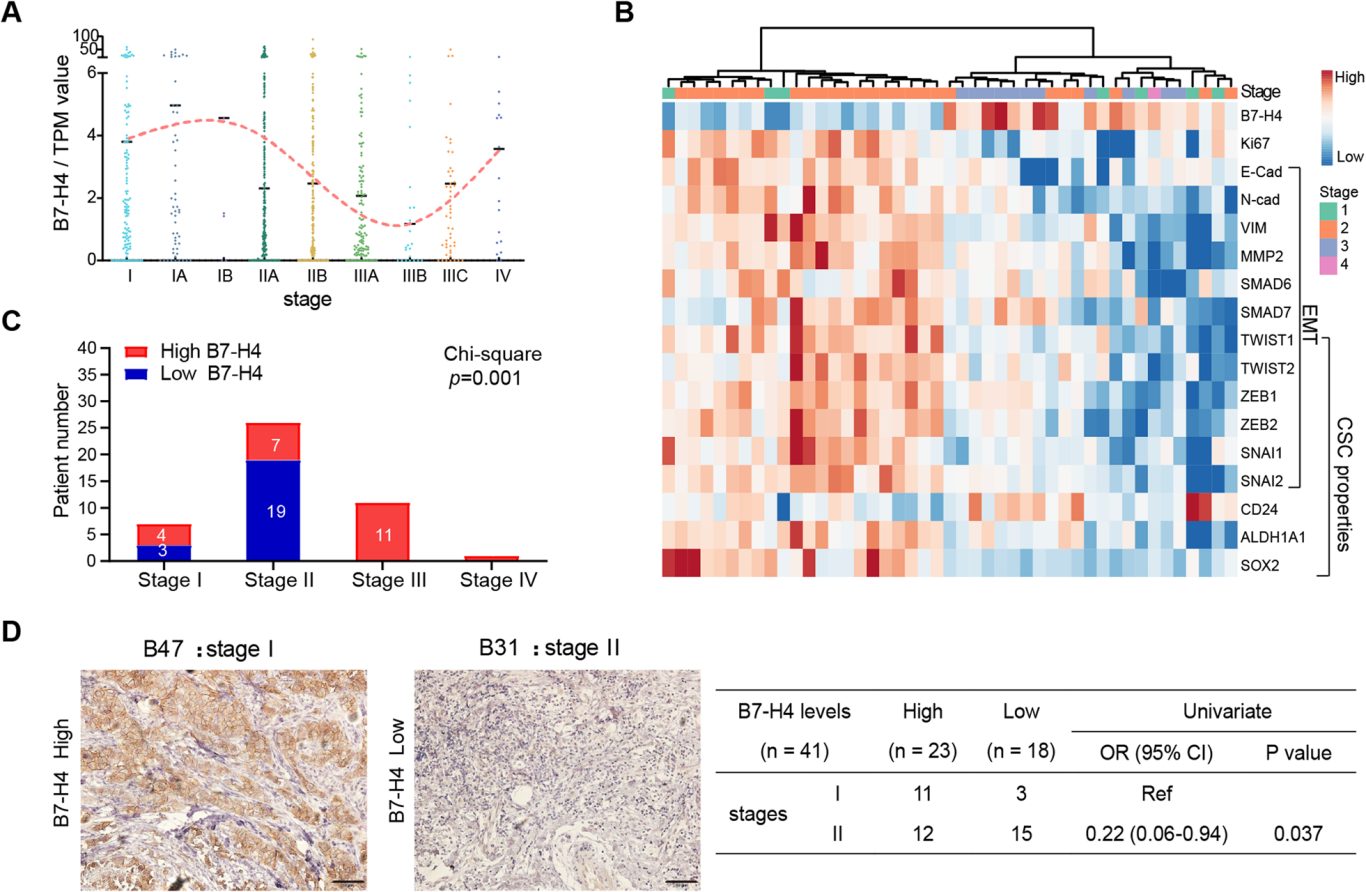
The fluctuation of B7-H4 expression levels among breast tumor stages might be associated with the immune escape of breast cancer cells in certain stages. **A** The expression levels of B7-H4 in terms of stages of breast cancer were evaluated according to the TCGA database (*n *= 932). **B** The selected 45 breast cancer patients were identified from the TCGA database with parallel expression patterns in which B7-H4 legitimizes EMT- and cell stemness-related genes. **C** The selected 45 patients were divided into high and low groups according to the population’s median value of B7-H4 expression. The patient number between the two groups was calculated in each stage. **D** B7-H4 protein expression levels in breast cancer tissues were detected by immunohistochemical staining (Scale bar = 50 μm), and the odd ratios of tumor stages between high versus low B7-H4 groups were computed using the chi-squared test. (*n *= 41)

**Fig. 7:**
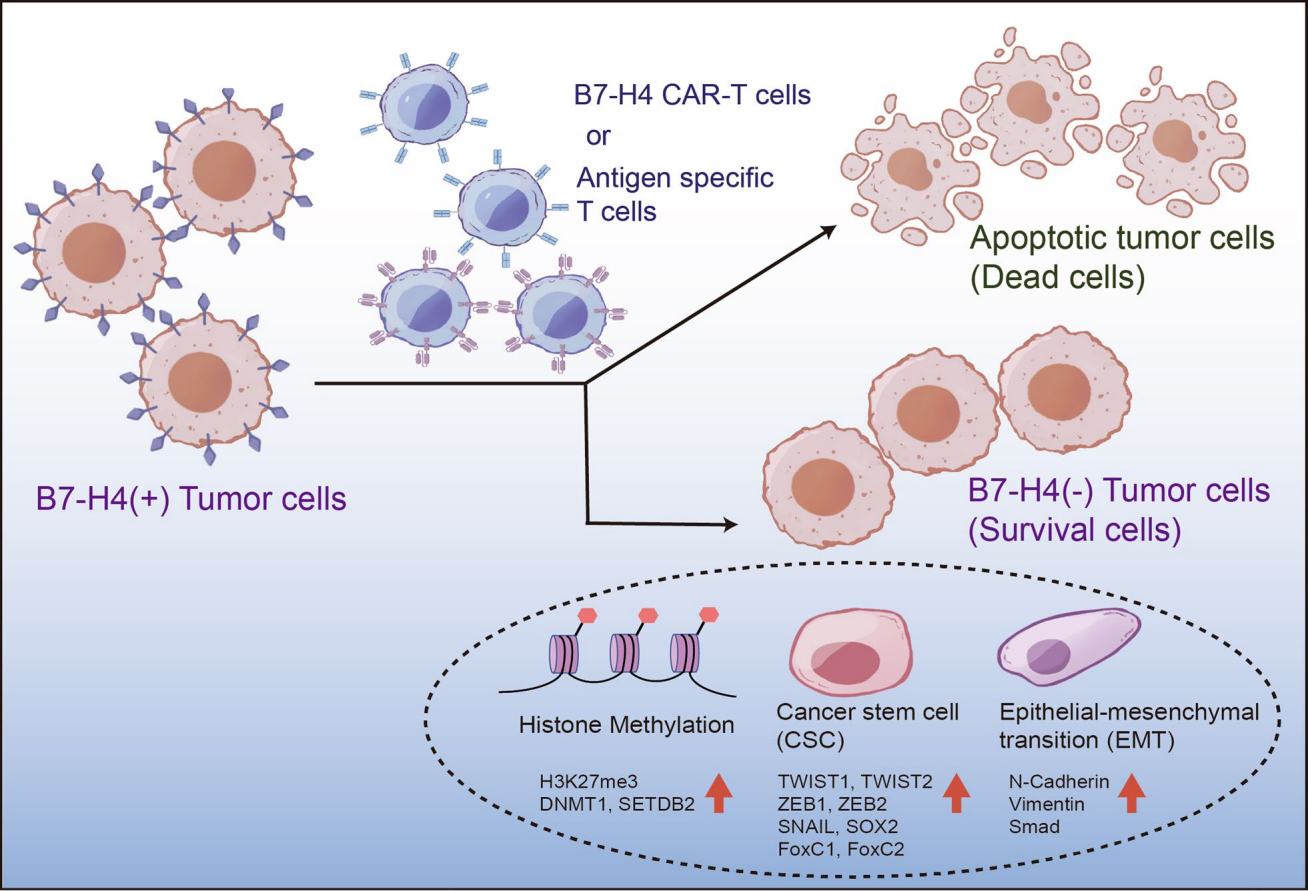
The potential mechanism of breast cancer cells escaping from T cell cytotoxicity. We describe a novel mechanism associated with immune escape of tumor cells in the context of CAR-T cell therapy and tumor progression. Based on our findings, some B7-H4 positive tumor cells will loss B7-H4 molecule when tumor cells encounter CAR-T cells target B7-H4. The absence of B7-H4 expression in tumor cells promotes epithelial–mesenchymal transition (EMT) and cancer stem cell (CSC) differentiation, resulting in tumor migration, metastasis and chemoresistance. Our study provided new insights into the role of EMT and CSC in tumor immune escape

The results above implied that downregulation of B7-H4 expression enhanced cancer cell metastasis from the primary tumor to distant organs, which might be a novel mechanism for the immune escape of breast cancer cells. To further demonstrate whether the circumstances exist, the TCGA BRCA database was accessed to investigate the correlation between B7-H4 expression levels and EMT characteristics in patients with breast cancer (*n *= 932). Notably, a gradual decrease in B7-H4 expression levels was found in breast cancer patients with stage II (*n *= 609) or stage III (*n *= 243) as compared to patients with stage I (*n *= 273) (Fig. 6A), and the similar trend was also observed in cervical cancer and ovarian cancer according to the TCGA database (Additional file 6: Fig. S6A and B). Moreover, our results from the TCGA BRCA databases revealed that the expression level of CD8 is slightly upregulated from AJCC stage II to stage III (Additional file 6: Fig. S6C), which is opposite to the change in B7-H4 expression from the same sample cohort. The reversed expression pattern of B7-H4 and CD8 is consistent with our previous results [15]. An inverse correlation between B7-H4- and EMT-related gene expression levels was found in a group of BRCA patients. The expression levels of EMT- and CSC-related genes were highly expressed in a group of low B7-H4 expression breast cancer patients (*n *= 22). On the other hand, these genes were decreased in high B7-H4 expression breast cancer patients (*n *= 23) (Fig. 6B). Importantly, low B7-H4 expression levels was observed in 19 (42.22%) stage II patients and only 3 (6.67%) stage I patients (Fig. 6C). Consistent with the results from TCGA BRCA, IHC staining showed that more percentages of stage II patients (15/27, 55.56%) were belonged to the groups of low B7-H4 protein expression, and only 3 of 14 (21.43%) of stage I patients was found in low B7-H4 groups (Fig. 6D). A decrease in OR according to the tumor stage classification was significantly observed in high B7-H4 breast cancer patients when compared to low B7-H4 breast cancer patients (Fig. 6D). In addition, the modified transwell assay shown in Fig. 1C revealed that the co-culture of SKBR3 cells with CAR-T cells drives the escape of a portion of tumor cells accompanied by lowering the B7-H4 expression. Taken together, our findings demonstrate that the upregulation of B7-H4 interferes with CTL-mediated tumor cytotoxicity, while the downregulation of B7-H4 expression level might be associated with the immune escape of breast cancer cells and contributes to tumor metastasis (Fig. 7).

## Discussion

Over the past few years, immune checkpoint inhibitor (ICI) targeting the PD-1/PD-L1 pathway has shown promising therapeutic effects in many cancers and has fundamentally changed the paradigm for the clinical management of cancer patients. Unfortunately, some patients fail to respond to PD-1/PD-L1 antibodies. Further study on the mechanism of other immune molecules is expected to develop new immunotherapy strategies. B7-H4, a member of the B7 family of immunoregulatory proteins, inhibits T cell proliferation and cytokine production. Notably, the B7-H4 protein is highly expressed in several tumors, including breast cancer, in combination with its low or absent protein expression in normal tissues, suggesting that B7-H4 is an attractive immunotherapeutic target for ICI’s [35]. The expression levels of B7-H4 have been reported to be associated with immunosuppression in the tumor microenvironment, tumor progression and poor prognosis, including urinary tract urothelial carcinoma, cervical cancer, colorectal cancer, lung cancer, ovarian cancer and breast cancer [16, 26, 36–39]. A mutually exclusive pattern of B7-H4 with PD-L1 expression has been demonstrated in some types of cancer, such as breast cancers, lung cancer and glioma [40–42], suggesting that some types of cancers might be preferentially undergoing B7-related immune evasion pathway.

Although the receptor on the T cell of B7-H4 remains unknown and controversial, it is generally known that cell surface-associated B7-H4 inhibits T cell activation and leads to a decrease in the number of CD4 and CD8 T cells [43, 44]. Our previous study also illustrated that high B7-H4 on breast cancer cells negatively correlated with CD8 T cell infiltration in tumor sites [15]. However, it is still unclear whether CTL could affect B7-H4 expression in tumor cells, contributing to the inverse correlation between B7-H4 and CD8 T cells. Our study shows that B7-H4 is downregulated in breast cancer cells after co-culture with CAR-T and the active T cells (Additional file 7). In addition, the absence of B7-H4 leads to an increase in breast cancer cell proliferation, migration and metastasis by promoting the progression of the cell cycle. Furthermore, B7-H4 can intervene in human breast cancer stem cell differentiation, EMT and chemoresistance.

Protection from CTL-mediated lysis has been reported to be associated with breast cancer cells undergoing EMT, wherein silencing of the WNT pathway coincides with hyperactivity of TGF-β signaling, EMT and acquisition of stemness properties [45]. Previous reports indicate that B7-H4 induces EMT and promotes cancer cell proliferation, invasion and stemness by using the temporary gene knockdown of cancer cell lines [38, 46–48]. Nevertheless, our results demonstrate that T cells-mediated downregulation of B7-H4 and B7-H4-KO breast cancer cells exhibits increased cell proliferation, migration and invasion. Cancer cells undergoing EMT processes implicate the loss of cell polarity and lead to the transformation of the mesenchymal phenotype, accompanied by regulation of E-cadherin and the upregulation of vimentin and/or N-cadherin. Meanwhile, the critical transcription factors, including *TWIST1*, *TWIST2*, *ZEB1*, *ZEB2*, *SNAI1* and *SNAI2*, participate in the EMT process, leading to cancer cell migration and invasion. Our results from RNA-seq analysis of these EMT- and CSC-related genes between different expression levels of B7-H4 breast cancer cells (KO vs. WT and WT vs. OE) further confirmed that B7-H4 deficiency promotes breast cancer cell growth, EMT and stemness.

Emerging evidence suggests a phenotypic and molecular signal pathway association between EMT and chemoresistance in several cancers, including breast cancer. In addition, tumor cells acquire stemness through the EMT process, secondary tumor-initiating and chemoresistance properties [29, 49]. The low expression of *CD24* and high expression of aldehyde dehydrogenase 1 family member A1 (*ALDH1A1*) may define subsets of breast cancer stem cells with enhanced migratory potential [50]. Our results further confirm the low expression of *CD24*, high expression of *ALDH1A1* and stem cell self-renew genes in B7-H4-KO breast cancer cells. In addition, the B7-H4 deficiency facilitates breast cancer cell growth, EMT and chemoresistance, with the opposite effects observed in the B7-H4 overexpression of breast cancer cells, including downregulation of migration and EMT critical genes such as vimentin and N-cadherin. Chemoresistance is a tremendous challenge in the progression and recurrence of breast cancer patients. We thus determined whether B7-H4 dysregulation may induce chemoresistance in breast cancer cells by evaluating the IC50 values for Doxorubicin [30], Oxaliplatin [31, 32], Fluorouracil [33] and Gefitinib [34], four commonly used chemotherapy in breast cancer patients. The significantly difference of IC50 was confirmed in SKBR3-KO or MDA-MB-231-OE cells, but not be seen in MCF7-OE cells (data not shown). These findings suggest that the loss of B7-H4 plays a crucial role in legitimizing breast cancer stem cell differentiation and EMT process and might at least in part mediate chemoresistance.

Epigenetic dysregulation is commonly observed in carcinogenesis and tumor progression and histone methylation is a critical epigenetic machinery in regulating cell gene transcription [51]. We have noticed in our transcriptomics analysis that B7-H4 knockout leads to a profound and broad influence on global gene transcription in SKBR3-KO cell lines, which prompted us to investigate whether B7-H4 may affect gene expression in breast cancer cells by epigenetic reprogramming. The enrichment analysis of differentially expressed genes showed that genes related to the upregulation of H3K27me3 were overrepresented in the B7-H4 KO cell line. Thus, we determined several candidate genes involved in DNA methylation and histone methylation and found a significant change in the expression of EZH2, the enzyme directly responsible for H3K27me3 generation after B7-H4 knockout. We also confirmed the difference in H3K27me3 levels between MDA-MB-231 WT and OE cells. These results support a potential link between B7-H4 dysregulation and histone methylation-mediated epigenetic reprogramming, as histone methylation is closely related to the subtyping and grading of breast cancers [52, 53]. The expression level of H3K27me3 has been shown to affect the expression of genes associated with EMT and stem cell potential to tolerate chemotherapy in breast cancer, leading to enhanced cancer cells metastasis and chemoresistance [54–56]. Given the molecular evidence from experiments and literature, we think that EZH2-H3K27me3 could be the possible regulators in mediating B7-H4-induced transcriptional alteration. Our results herein demonstrate that B7-H4 deficiency promotes H3K27me3 but H3K9me3, resulting in enhanced breast cancer stem cells and the potential for epigenetic reprogramming.

To better elucidate our surprisingly novel findings into the role of B7-H4 in breast cancer cells, the tumor microenvironment involved with immune cells should certainly be considered in factual circumstances. We analyzed the expression levels of B7-H4 using the TCGA BRCA database profiling 932 breast tumor patients. The data demonstrate that the expression levels of B7-H4 are significantly downregulated from AJCC stage II to stage III. Our results further confirm the inverse expression levels of B7-H4 with EMT- and CSC-related genes in a group of breast cancer patients. The results strongly suggest that downregulation of B7-H4 leads to breast tumor growth and an invasion of the nearby lymph nodes undergoing the EMT process but also indicate that the breast tumor immune microenvironment plays a critical role in the impairment of immune system responses in developing tumor progression. Considering the widespread expression of B7-H4 in different cancer types and low expression in normal tissues, B7-H4 is a reasonable target for the blockades, in which monotherapy such as CD3 bispecific antibody (BsAb) has proven effective in mouse models [57]. However, based on our findings, we should be carefully considered for application on patients of different breast cancer stages when using CAR-T or BsAb target to B7-H4 for destruction by interfering tumor immune microenvironment and incorporating with appropriate chemotherapy.

## Conclusions

In contrast to the previously described B7-H4, the surprising novel results from our study demonstrate that the absence of B7-H4 promotes breast cancer cell growth, EMT and chemoresistance. In the tumor immune microenvironment involved with immune cell interaction, the timeline effect of B7-H4 shows the critical function of legitimizing human breast cancer cell immune escape via EMT and stemness. These shreds of evidence strongly suggest that B7-H4 promoting tumor progression is required to harmonize the negative regulation of CTLs in the tumor microenvironment.

### Supplementary Information

**Additional file 1: Fig. S1. **The decrease in B7-H4 expression was found in MD-MBA-468 cells co-cultured with B7-H4 CAR-T cells.

**Additional file 2: Fig. S2. **B7-H4 deficiency increased cell proliferation and cell cycle exoneration in breast cancer cells.

**Additional file 3: Fig. S3.** B7-H4 deficiency facilitates EMT and stemness characteristics of human breast cancer cells.

**Additional file 4: Fig. S4**. B7-H4 deficiency promotes breast cancer cell stemness and chemoresistance.

**Additional file 5: Fig. S5**. Lung metastasis of different breast cancer cells mouse model.

**Additional file 6: Fig. S6**. The expression levels of B7-H4 in terms of stages of CESC and OVCA and the expression levels of CD8 in terms of stages of BRCA.

**Additional file 7**: Full western blot image corresponding to Fig. 1H, Fig. S1G, Fig. 2B, Fig. S2D, Fig. S3D, and Fig. S4F.

## Data Availability

All data generated or analyzed during this study are available from the corresponding author on reasonable request.

## Abbreviations

EMT: Epithelial-to-mesenchymal transition
B7-H4: B7 homology 4
TIME: Tumor immune microenvironment
KO: Knockout
OE: Overexpression
WT: Wild type
CARs: Chimeric antigen receptors
TCGA: The Cancer Genome Atlas
BRCA: Breast cancer
PD-L1: Programmed death-ligand 1
PD-1: Programmed death-1
CTLA-4: Cytotoxic T-lymphocyte-associated protein 4
CTL: Cytotoxic T-Lymphocyte
ER: Estrogen receptor
CFSE: Carboxyfluorescein diacetate succinimidyl ester
IHC: Immunohistochemistry
GSEA: Gene set enrichment analysis
HR: Hormone receptor
HER2: Human epidermal growth factor receptor 2
TNBC: Triple-negative breast cancer
CSC: Cancer stem cell
ICI: Immune checkpoint inhibitor
