# Supplementary material for: The loss of B7-H4 expression in breast cancer cells escaping from T cell cytotoxicity contributes to epithelial-to-mesenchymal transition
Source: Breast Cancer Res. 2023 Oct 4;25:115. doi: 10.1186/s13058-023-01721-5 (PMC10548745; doi:10.1186/s13058-023-01721-5)
Supplement: Supplementary file 1 — Additional file 1: Fig. S1. The decrease in B7-H4 expression was found in MD-MBA-468 cells co-cultured with B7-H4 CAR-T cells. [file 13058_2023_1721_MOESM1_ESM.docx]

**Additional file 1**


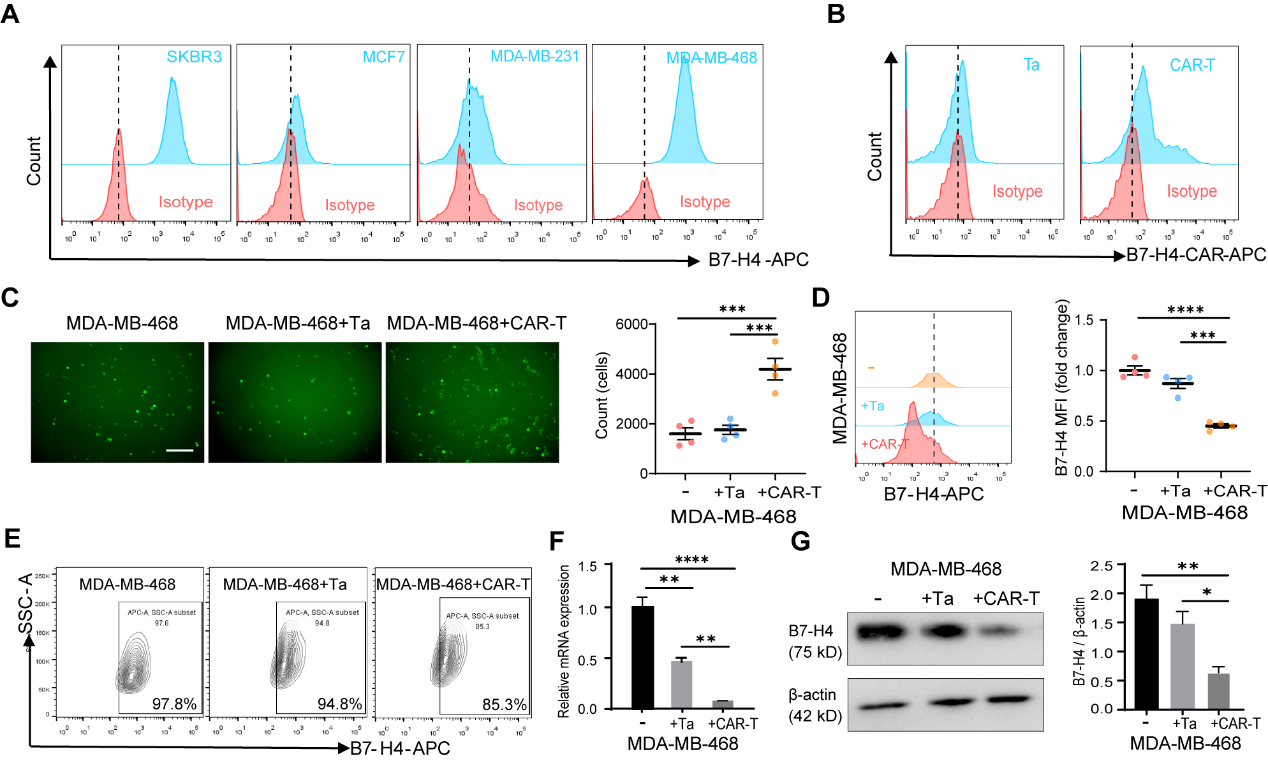


**Additional file 1: Fig.S1 The decrease of B7-H4 expression was found in MD-MBA-468 cells co-culture with B7-H4 CAR-T cells.**

**A** The expression levels of B7-H4 were analyzed by Flow Cytometry in various human breast cancer cell lines. **B** Identification of B7-H4 CAR infected primary human T cells determined by flow cytometry. **C** T cell-induced escape of CFSE- labelled MD-MBA-468 cells into the lower chamber in the co-cultured transwell model was observed by microscopy (left graph) and quantified by flow cytometry analysis (right graph). Scale bar = 100 μm. **D** B7-H4 expression levels of MD-MBA-468 cells in the lower chamber of different groups were quantified by flow cytometry analysis. **E-G** B7-H4 expression levels of residual living MD-MBA-468 cells were analyzed by flow cytometry (**E**), qPCR (**F**) and western blot (**G**) after co-culture with non-specific activated T cells and B7-H4 CAR-T cells, respectively. The data represent the mean ± SEM from three independent experiments and statistical significance was determined by one-way ANOVA. * p < 0.05, ** p < 0.01, *** p < 0.001, **** p < 0.0001. Ta: activated T cells.
