# Supplementary material for: The loss of B7-H4 expression in breast cancer cells escaping from T cell cytotoxicity contributes to epithelial-to-mesenchymal transition
Source: Breast Cancer Res. 2023 Oct 4;25:115. doi: 10.1186/s13058-023-01721-5 (PMC10548745; doi:10.1186/s13058-023-01721-5)
Supplement: Supplementary file 2 — Additional file 2: Fig. S2. B7-H4 deficiency increased cell proliferation and cell cycle exoneration in breast cancer cells. [file 13058_2023_1721_MOESM2_ESM.docx]

**Additional file 2**

**
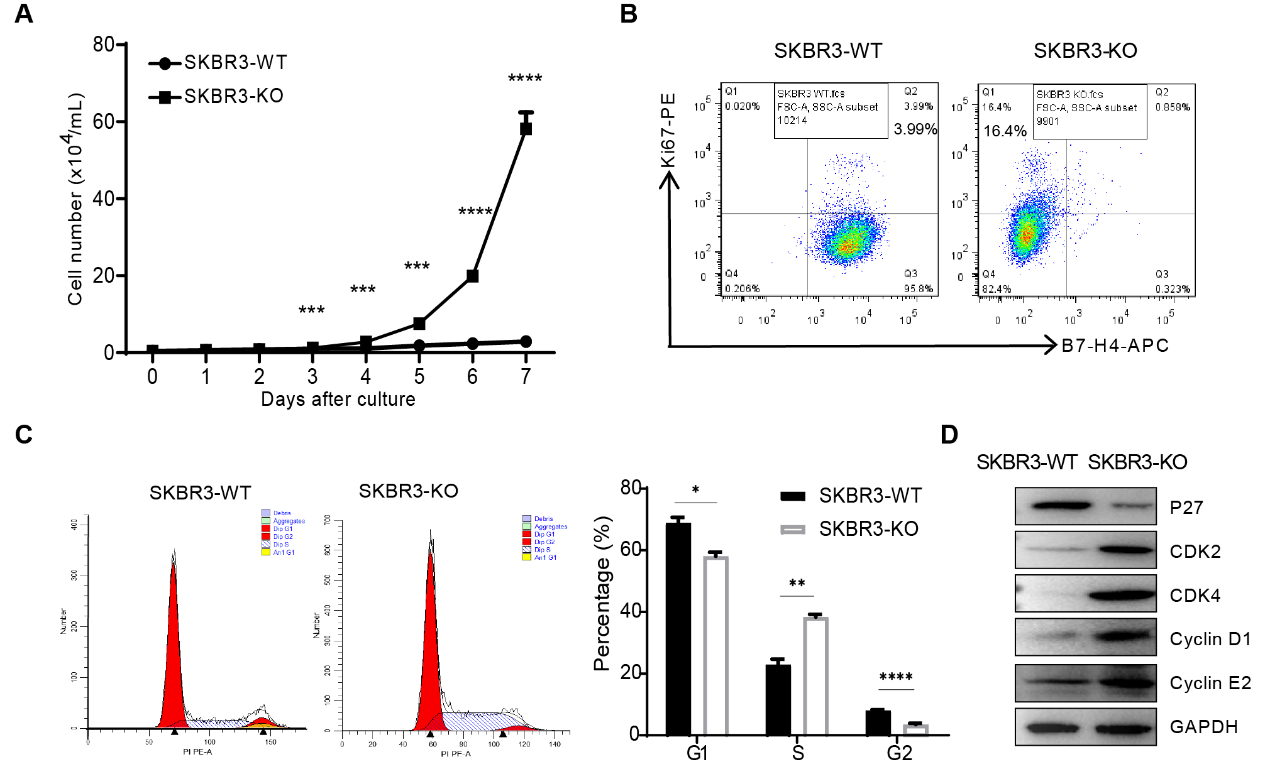
**

**Fig. S2. B7-H4 deficiency increased cell proliferation and cell cycle exoneration in breast cancer cells.**

**A** The growth rates of SKBR3-WT and SKBR3-KO cells were investigated by cell number counting. **B** The expression of Ki-67 in SKBR3-WT and SKBR3-KO cells was analyzed by flow cytometry. **C** Cell cycle analysis and quantification of cell percentages in each phase were using flow cytometry. **D** The cell cycle regulators were evaluated by immunoblotting. Data are represented as mean ± SEM of three independent experiments and statistical significance was determined by a two-tailed unpaired *t*-test. (* p < 0.05, ** p < 0.01, *** p < 0.001, **** p< 0.0001).
