# Supplementary material for: The loss of B7-H4 expression in breast cancer cells escaping from T cell cytotoxicity contributes to epithelial-to-mesenchymal transition
Source: Breast Cancer Res. 2023 Oct 4;25:115. doi: 10.1186/s13058-023-01721-5 (PMC10548745; doi:10.1186/s13058-023-01721-5)
Supplement: Supplementary file 3 — Additional file 3: Fig. S3. B7-H4 deficiency facilitates EMT and stemness characteristics of human breast cancer cells. [file 13058_2023_1721_MOESM3_ESM.docx]

**Additional file 3**


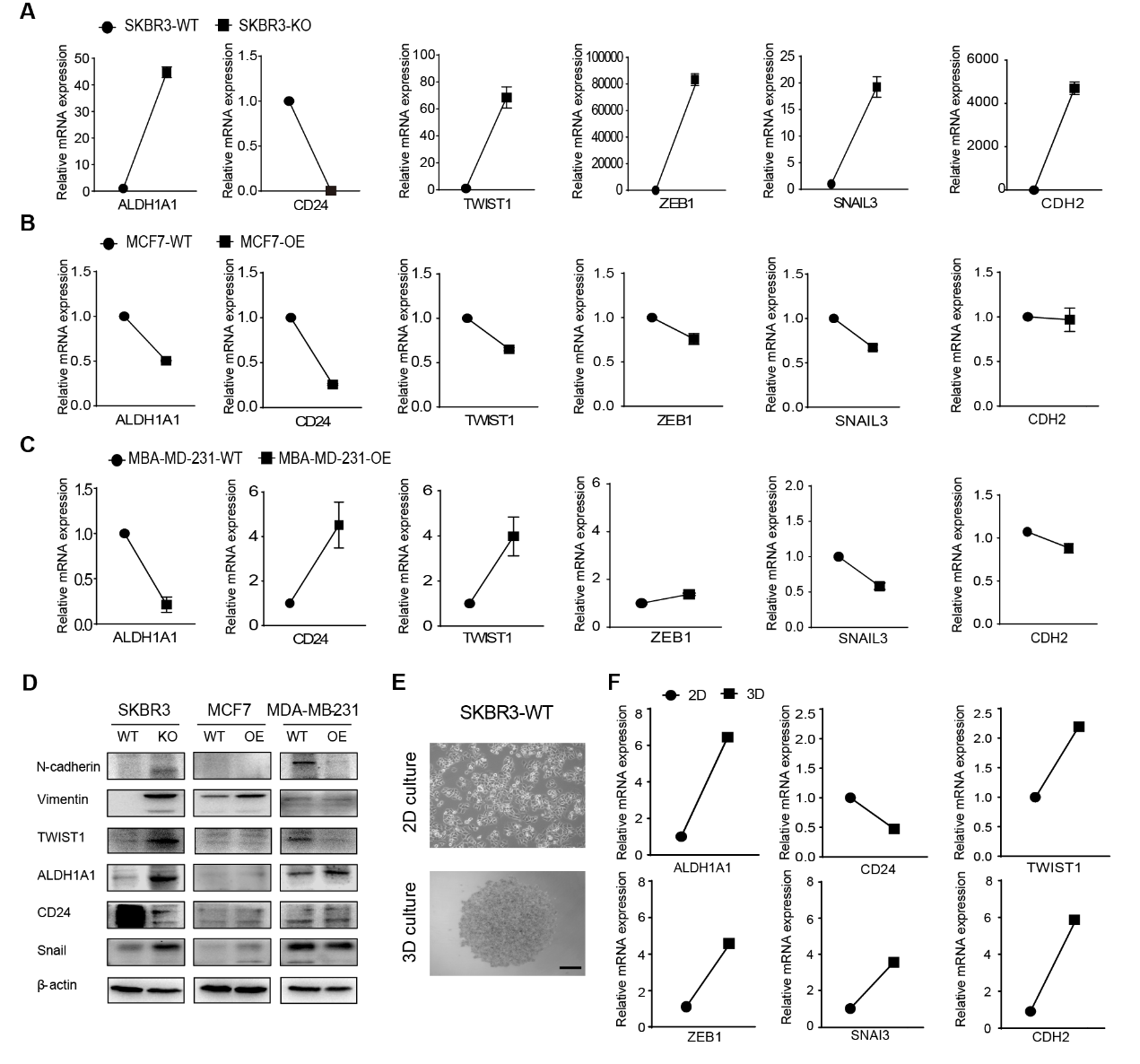


**Fig. S3.** **B7-H4 deficiency facilitates EMT and stemness characteristics of human breast cancer cells.**

**A-C** The mRNA expression of EMT and cell stemness markers in SKBR3 (WT and KO), MCF7 (WT and OE), and MDA-MB-231 (WT and OE) cells were investigated by qPCR. **D** The expression of EMT and cell stemness markers in SKBR3 (WT and KO), MCF7 (WT and OE), and MDA-MB-231 (WT and OE) cells were analyzed by Western blot. **E** Morphological changes of SKBR3-WT cells in 2D and 3D spheroids cultures are monitored respectively. Scale bar = 50μm. **F** The mRNA expression of cell stemness-related genes was characterized in 2D and 3D spheroids cultures. Bars are means ± SEM (n=5).
