# Supplementary material for: The loss of B7-H4 expression in breast cancer cells escaping from T cell cytotoxicity contributes to epithelial-to-mesenchymal transition
Source: Breast Cancer Res. 2023 Oct 4;25:115. doi: 10.1186/s13058-023-01721-5 (PMC10548745; doi:10.1186/s13058-023-01721-5)
Supplement: Supplementary file 4 — Additional file 4: Fig. S4. B7-H4 deficiency promotes breast cancer cell stemness and chemoresistance. [file 13058_2023_1721_MOESM4_ESM.docx]

**Additional file 4**


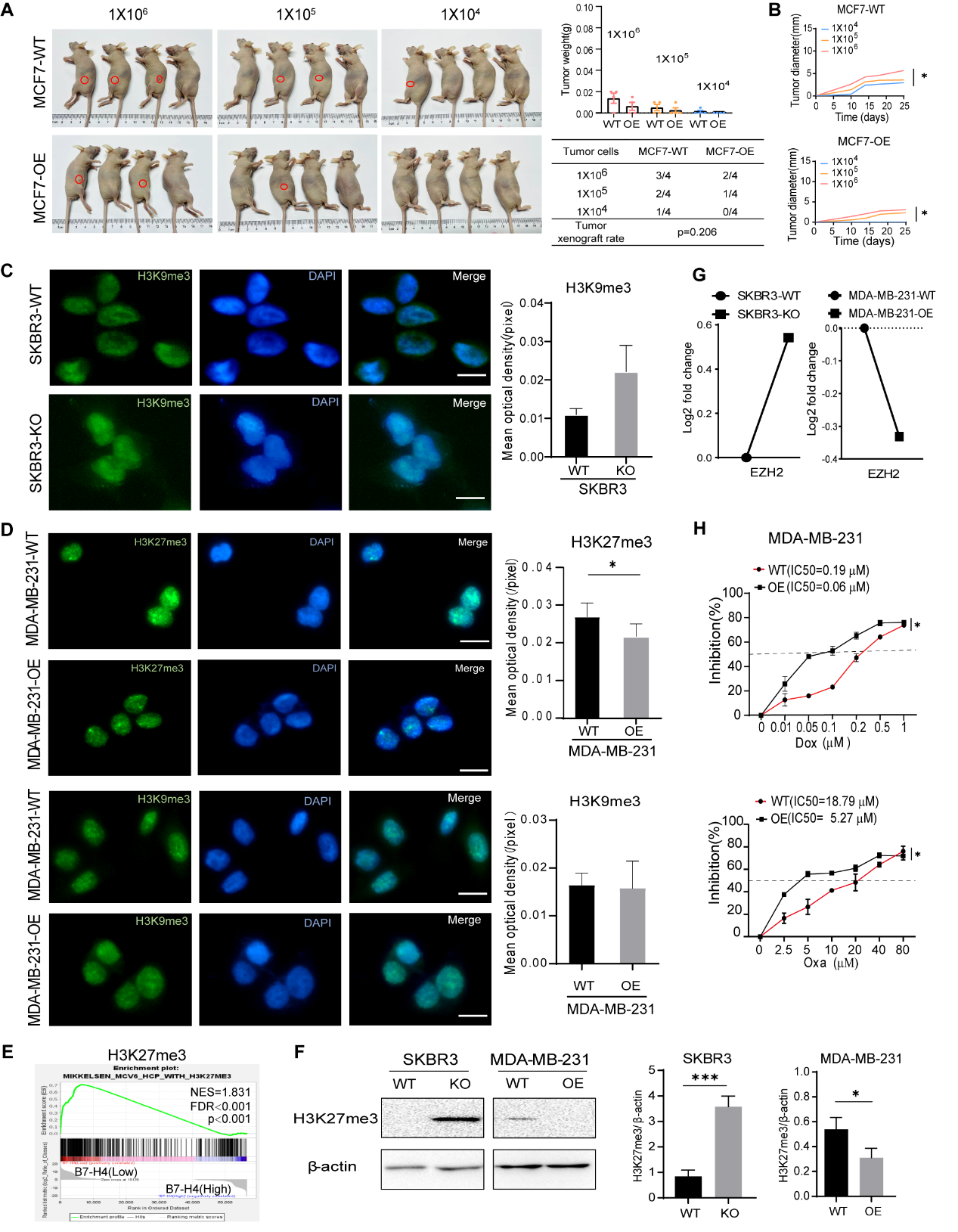


**Additional file 4: Fig.S4 B7-H4 deficiency promotes breast cancer cell stemness and chemoresistance.**

**A** *In vivo* tumorigenicity of breast cancer cells were performed in the subcutaneous xenograft mouse model at limiting dilutions. Tumors were isolated from nude mice 25 days postinoculation. **B** Tumor diameters in MCF-7 WT and OE were measured every other day after xenografting. **C** Immunofluorescence reveals the expression levels of H3K9me3 in SKBR3 (WT and KO) cells. **D** The expression levels of H3K27me3 and H3K9me3 in MDA-MB-231 (WT and OE) cells were detected using immunofluorescence. Scale bar = 10 μm. **E** The H3K27me3 enriched pathways based on differentially expressed genes in B7-H4 High group (SKBR3-WT and MDA-MB-231-OE cells) and B7-H4 Low group (SKBR3-KO and MDA-MB-231-WT cells) was performed by the GSEA analysis. **F** Western blot investigated the expression levels of H3K27me3 in SKBR3 and MDA-MB-231 cells. **G** The methyltransferase for H3K27me3 were examined by qPCR. **H** Quantifying IC50 values of MDA-MB-231 WT and OE cells for Dox (Doxorubicin), Oxa (Oxaliplatin) by MTT assay. Data are represented as mean ± SEM (n=5) of three independent experiments and statistical significance was determined by two-tailed unpaired t-test. Statistical assessment of the tumor growth curves was determined by one-way ANOVA (B). (* p < 0.05, *** p < 0.001).
