# Supplementary material for: The loss of B7-H4 expression in breast cancer cells escaping from T cell cytotoxicity contributes to epithelial-to-mesenchymal transition
Source: Breast Cancer Res. 2023 Oct 4;25:115. doi: 10.1186/s13058-023-01721-5 (PMC10548745; doi:10.1186/s13058-023-01721-5)
Supplement: Supplementary file 5 — Additional file 5: Fig. S5. Lung metastasis of different breast cancer cells mouse model. [file 13058_2023_1721_MOESM5_ESM.docx]

**Additional file 5**

**
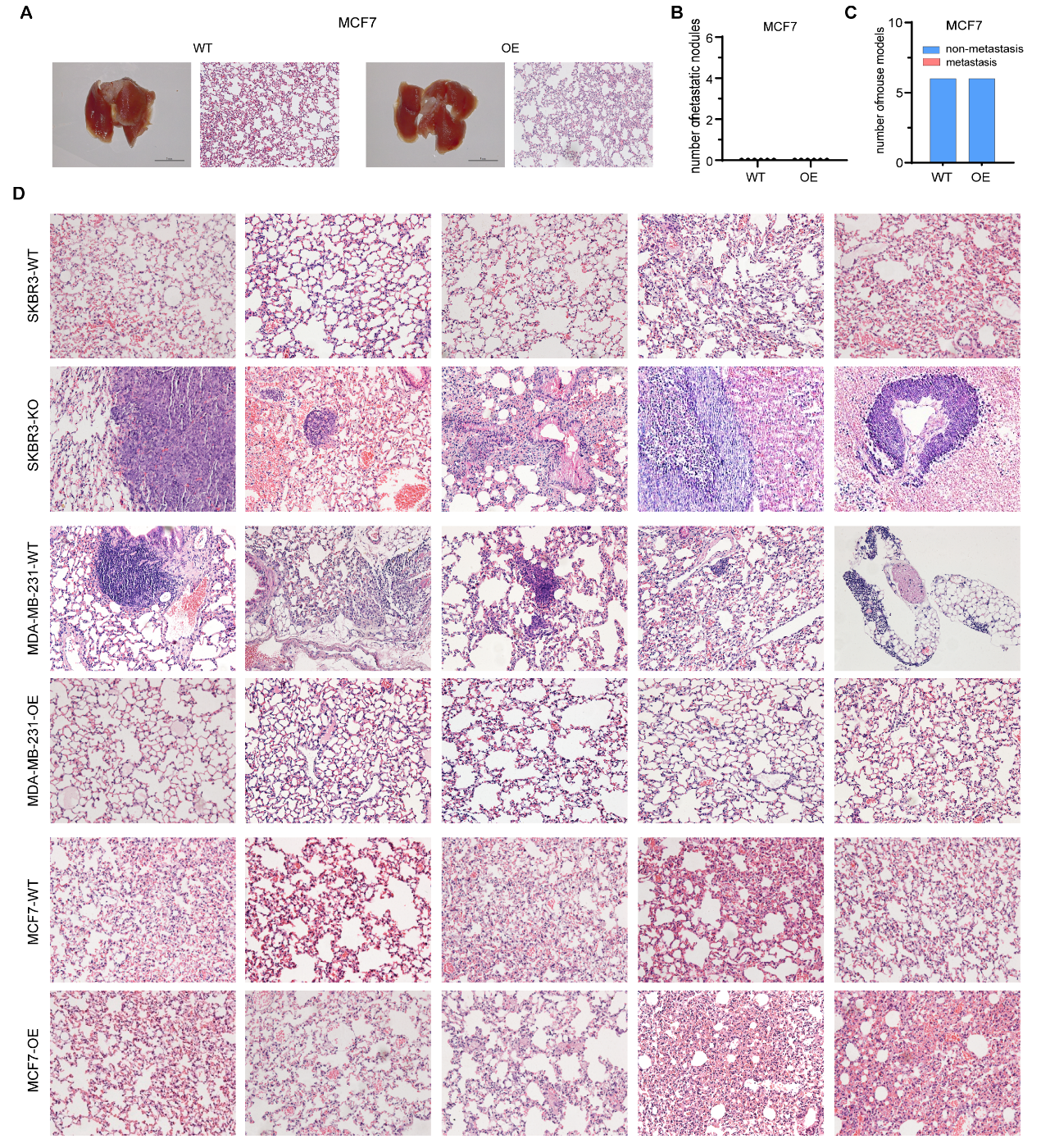
**

**Fig. S5. Lung metastasis of different breast cancer cells mouse model**.

(A) The representative lung tissue images (Scale bar = 5 mm) and HE staining (Scale bar = 100 μm) of MCF-7 (WT and OE) were shown. (B) The number of metastatic nodules in representative lung tissue were calculated. (C) The total number of mice with and without metastatic lung tumors (metastasis and non-metastasis) in each group (n = 5) were calculated. (D) The HE staining images of SKBR3 (WT and KO), MDA-MB-231 (WT and OE), and MCF7 (WT and OE) were shown. Scale bar = 50 μm.
