# Supplementary material for: The loss of B7-H4 expression in breast cancer cells escaping from T cell cytotoxicity contributes to epithelial-to-mesenchymal transition
Source: Breast Cancer Res. 2023 Oct 4;25:115. doi: 10.1186/s13058-023-01721-5 (PMC10548745; doi:10.1186/s13058-023-01721-5)
Supplement: Supplementary file 6 — Additional file 6: Fig. S6. The expression levels of B7-H4 in terms of stages of CESC and OVCA and the expression levels of CD8 in terms of stages of BRCA. [file 13058_2023_1721_MOESM6_ESM.docx]

**Additional file 6**


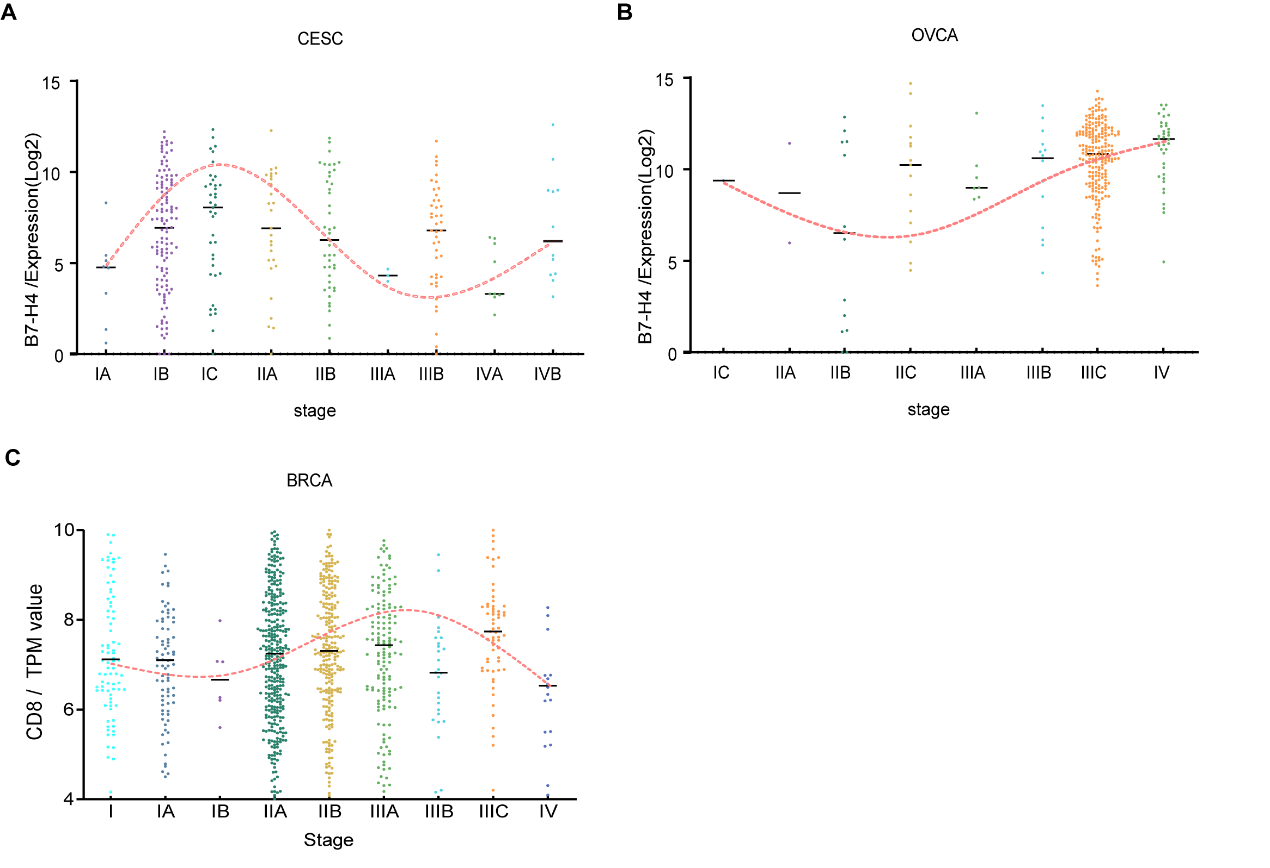


**Additional file 6: Fig.S6** **The expression levels of B7-H4 in terms of stages of CESC and OVCA and the expression levels of CD8 in terms of stages of BRCA.**

Based on the data from TCGA database, B7-H4 expression levels were evaluated in different stages of (**A**) Cervical cancer (CESC) and (**B**) Ovarian cancer (OVCA). **C** The expression levels of CD8 in terms of stages of breast cancer (BRCA) were evaluated according to the TCGA database.
