# Supplementary material for: The loss of B7-H4 expression in breast cancer cells escaping from T cell cytotoxicity contributes to epithelial-to-mesenchymal transition
Source: Breast Cancer Res. 2023 Oct 4;25:115. doi: 10.1186/s13058-023-01721-5 (PMC10548745; doi:10.1186/s13058-023-01721-5)

Additional file 7: Full western blot image corresponding to Fig.1H, Fig.S1G, Fig.2B, Fig.S2D, Fig.S3D, and Fig.S4F.

M:Marker

Fig.1H-(B7-H4)

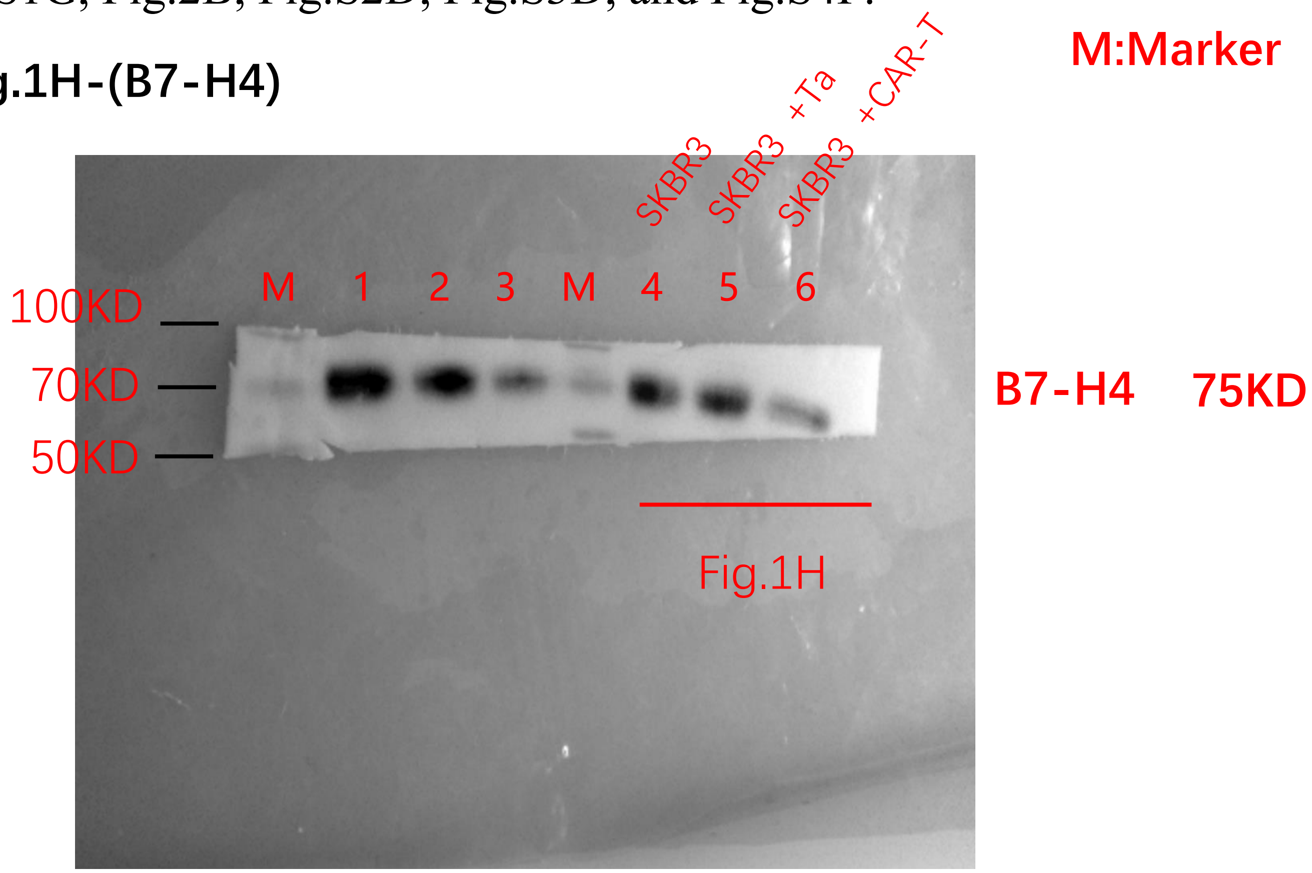

Fig.1H-(actin)

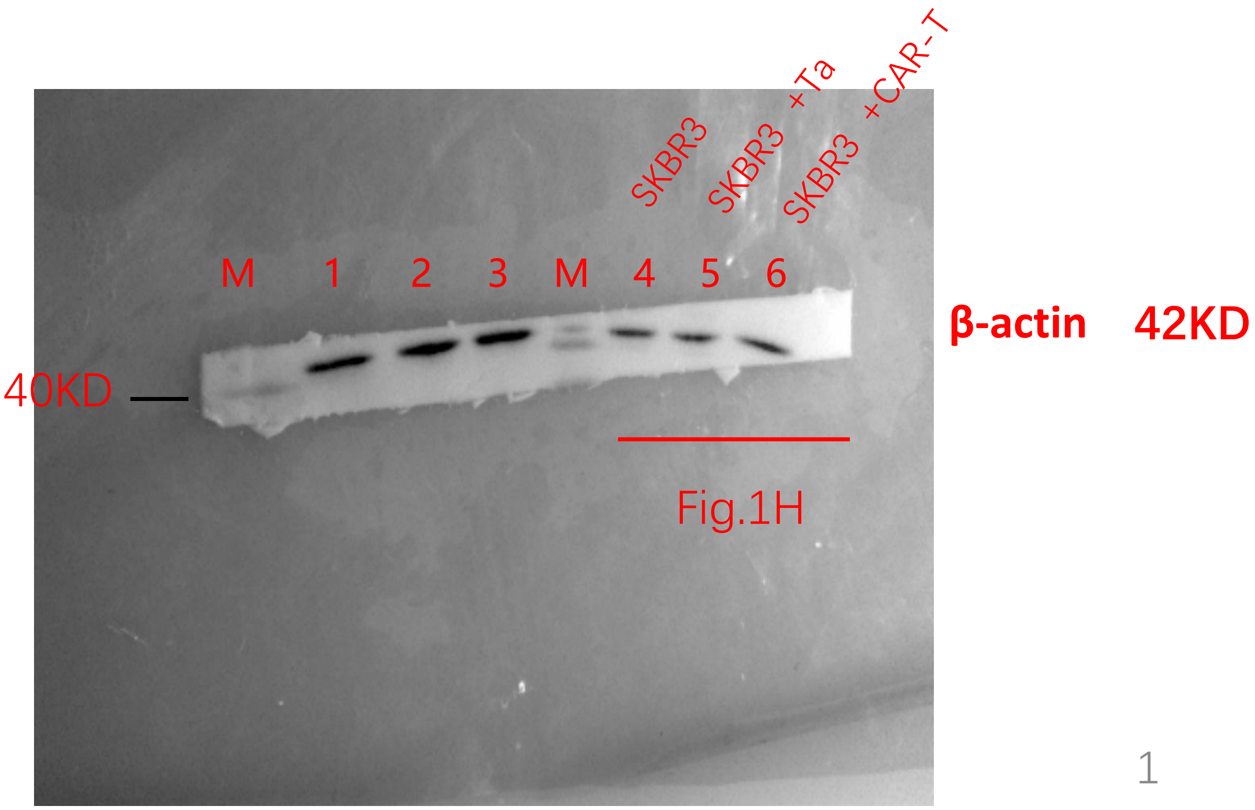

M:Marker

Fig.2B-Left (B7-H4)

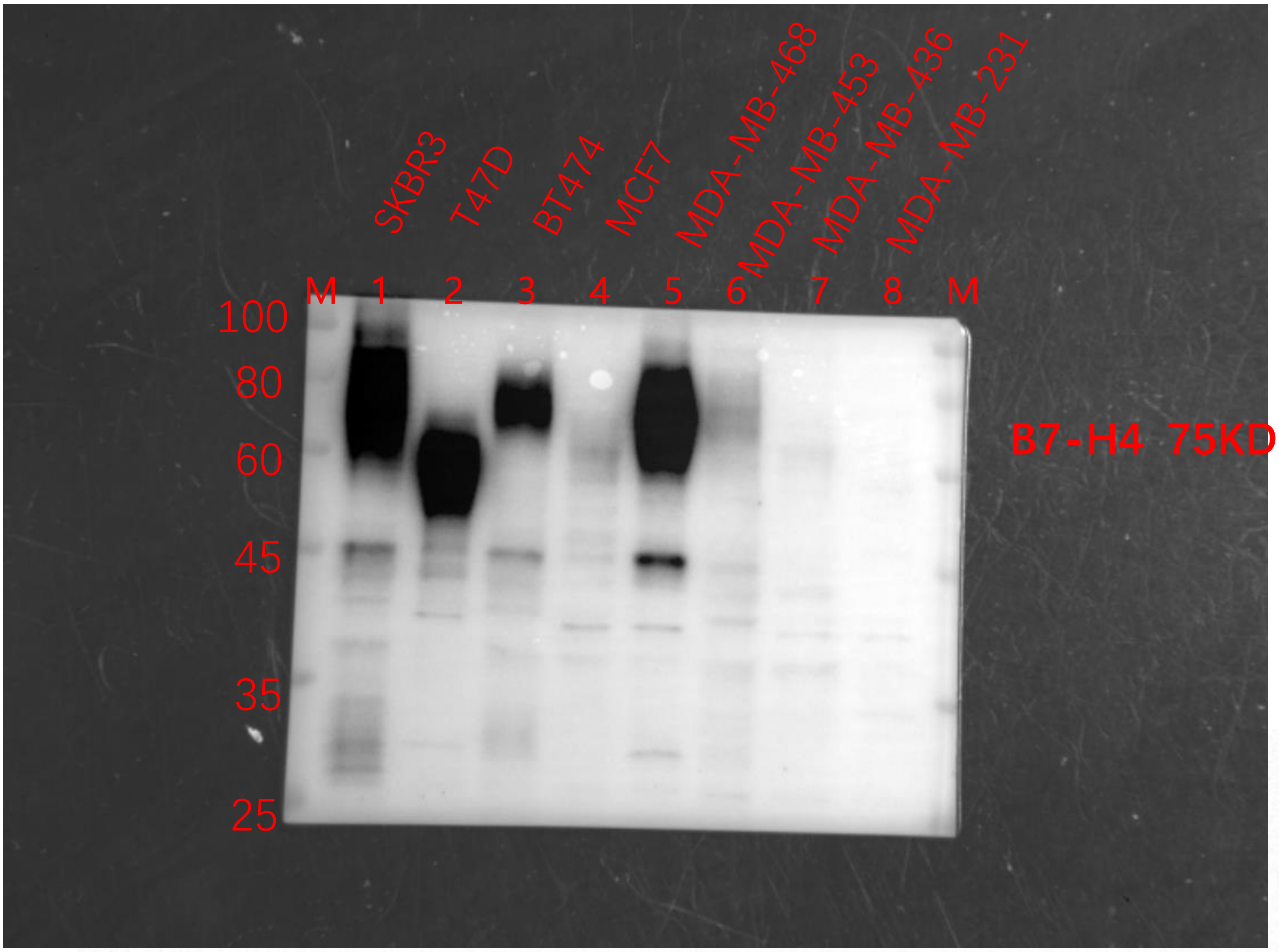

Fig.2B-Left (actin)

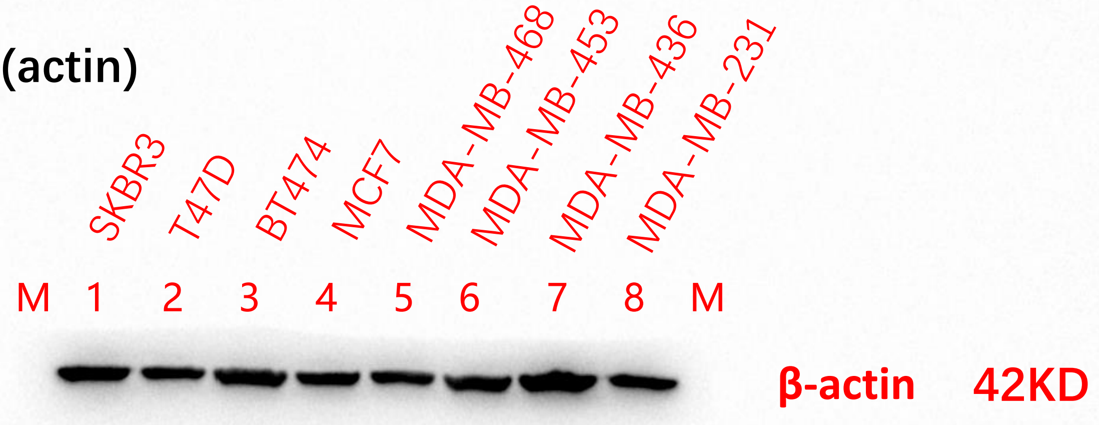

M:Marker

Fig.2B-Right (B7-H4)

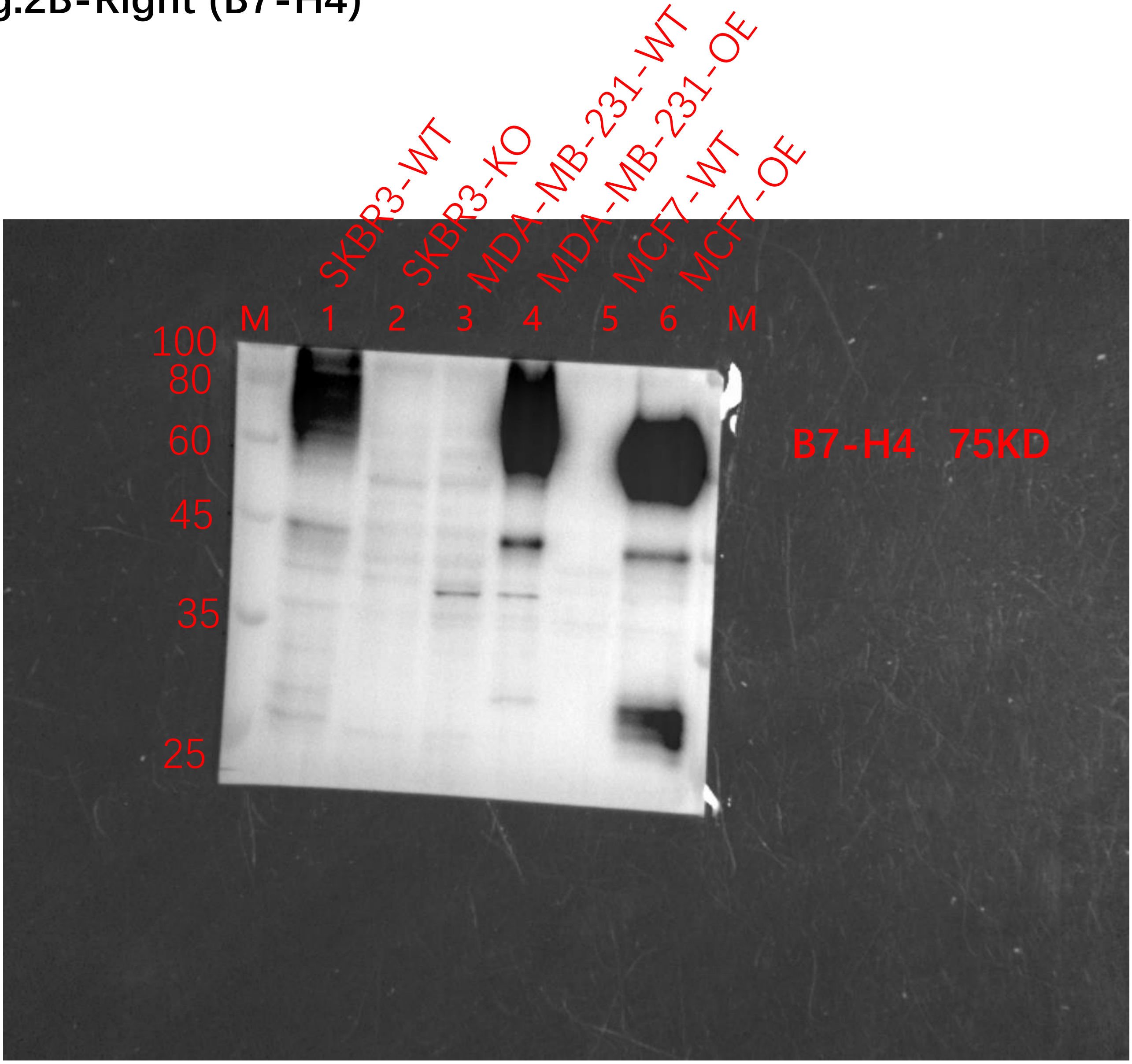

Fig.2B-Right (actin)

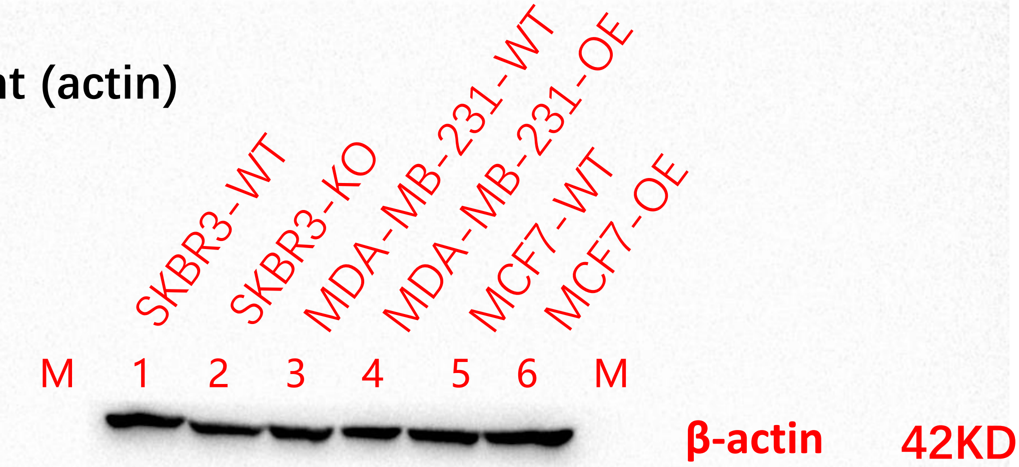

M:Marker

Fig.S1G-(B7-H4)

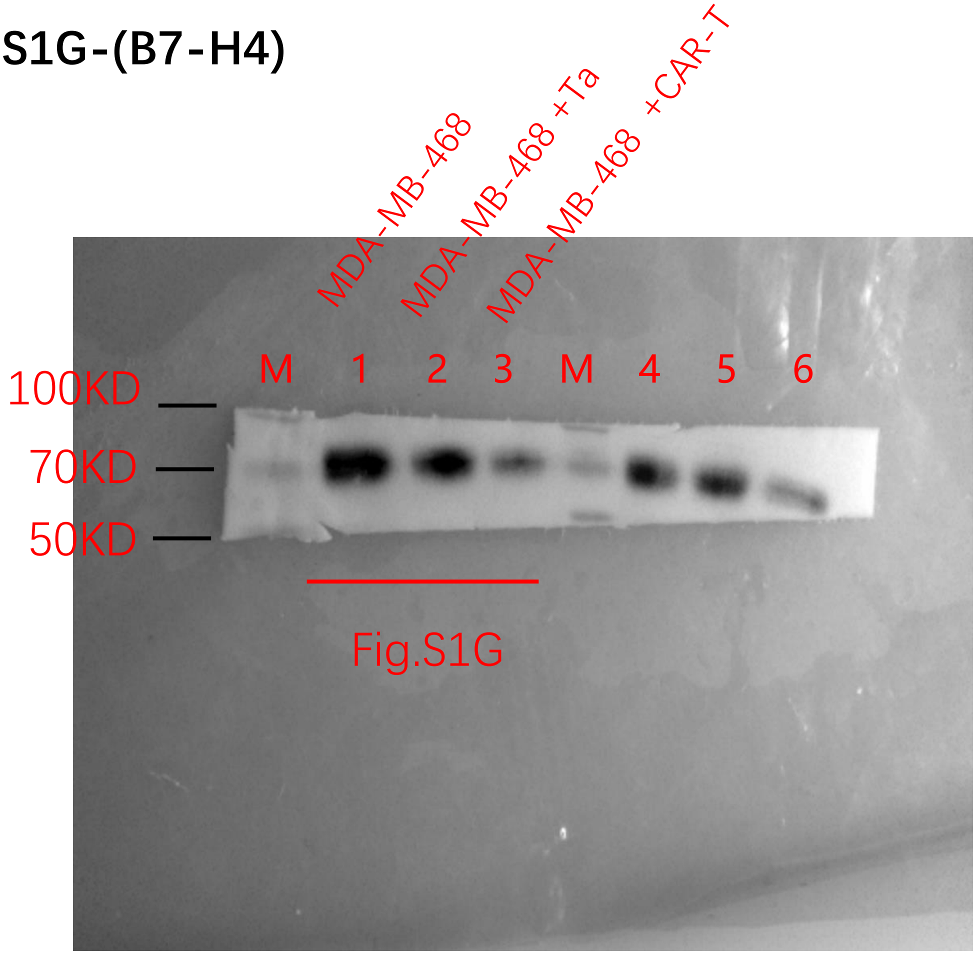

B7-H4 75KD

Fig.S1G-(actin)

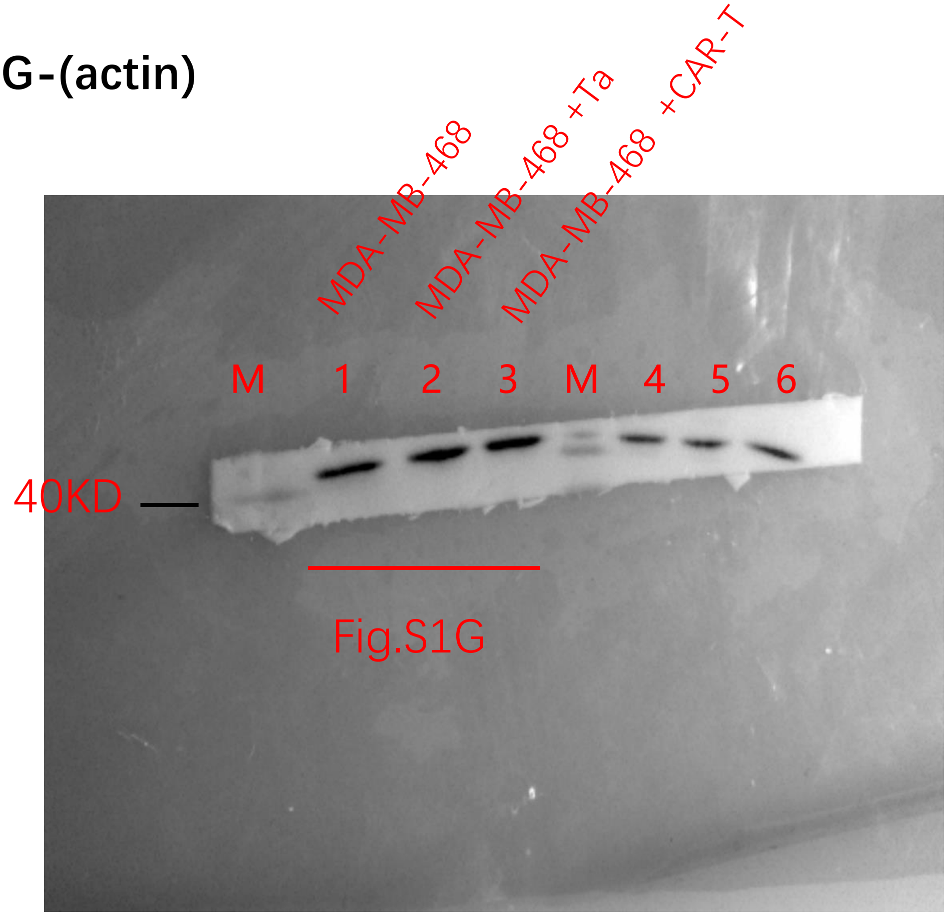

β-actin 42KD

Fig.S2D

M:Marker

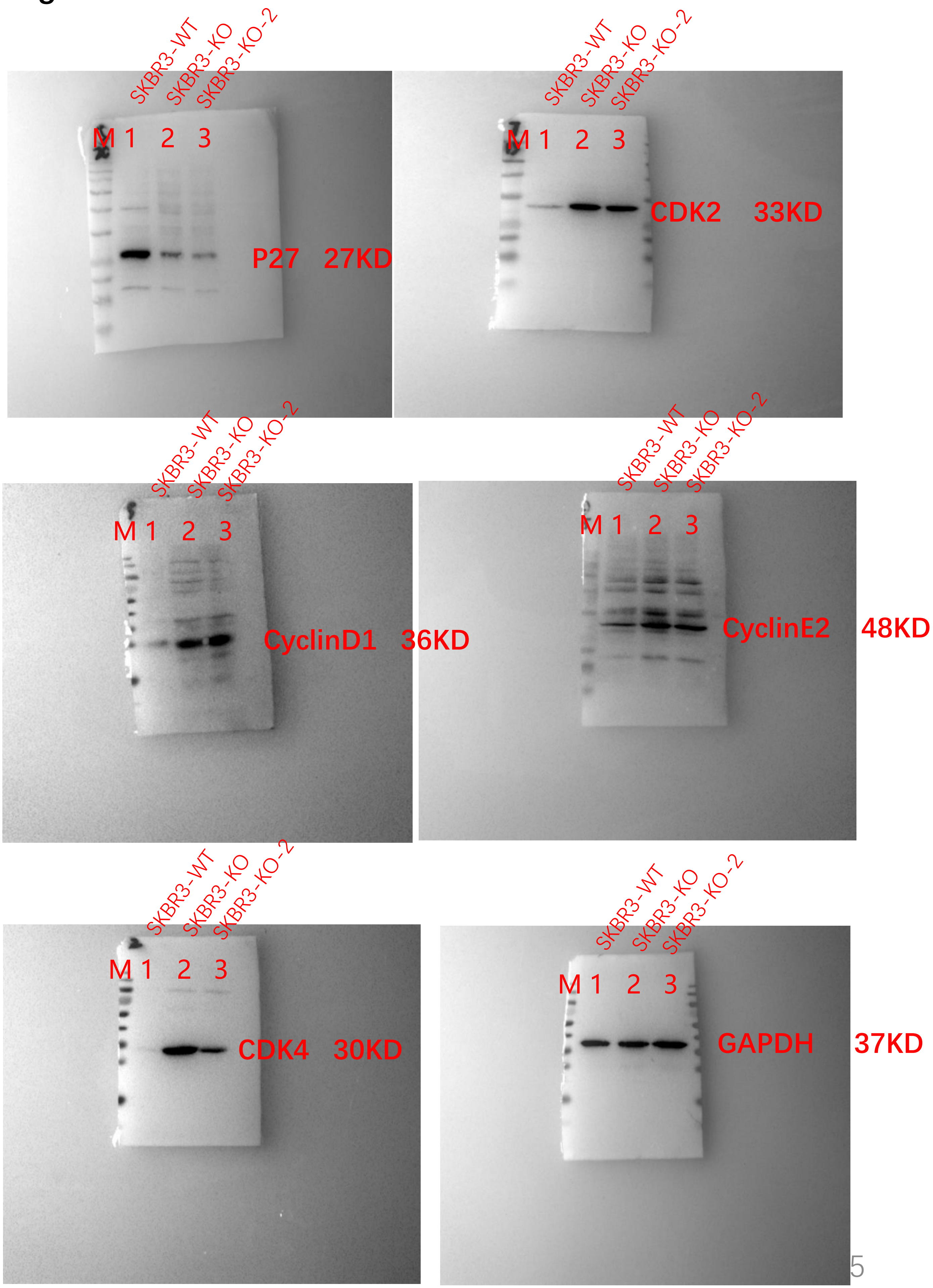

M:Marker

Fig.S3D-Snail

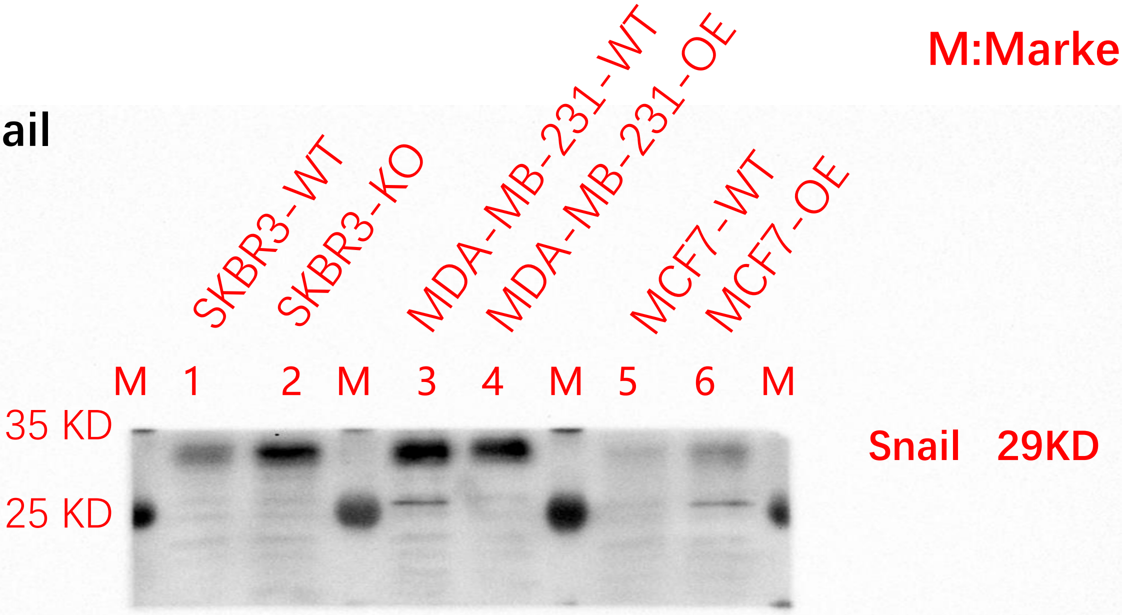

Fig.S3D-ALDH1A1

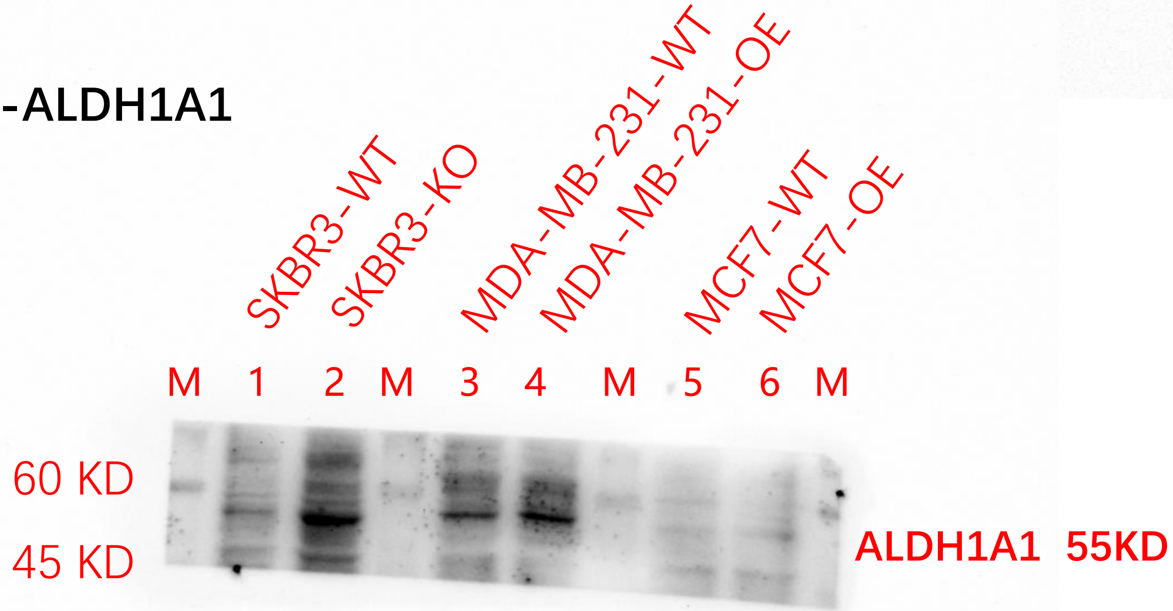

β-actin

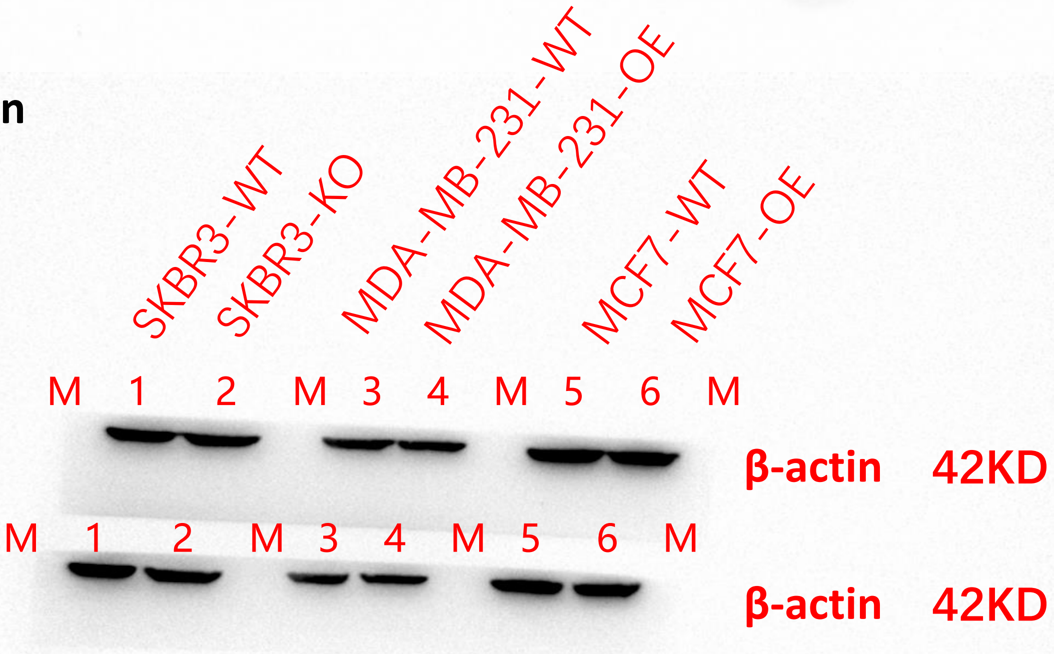

M:Marker

Fig.S3D-N-cadherin

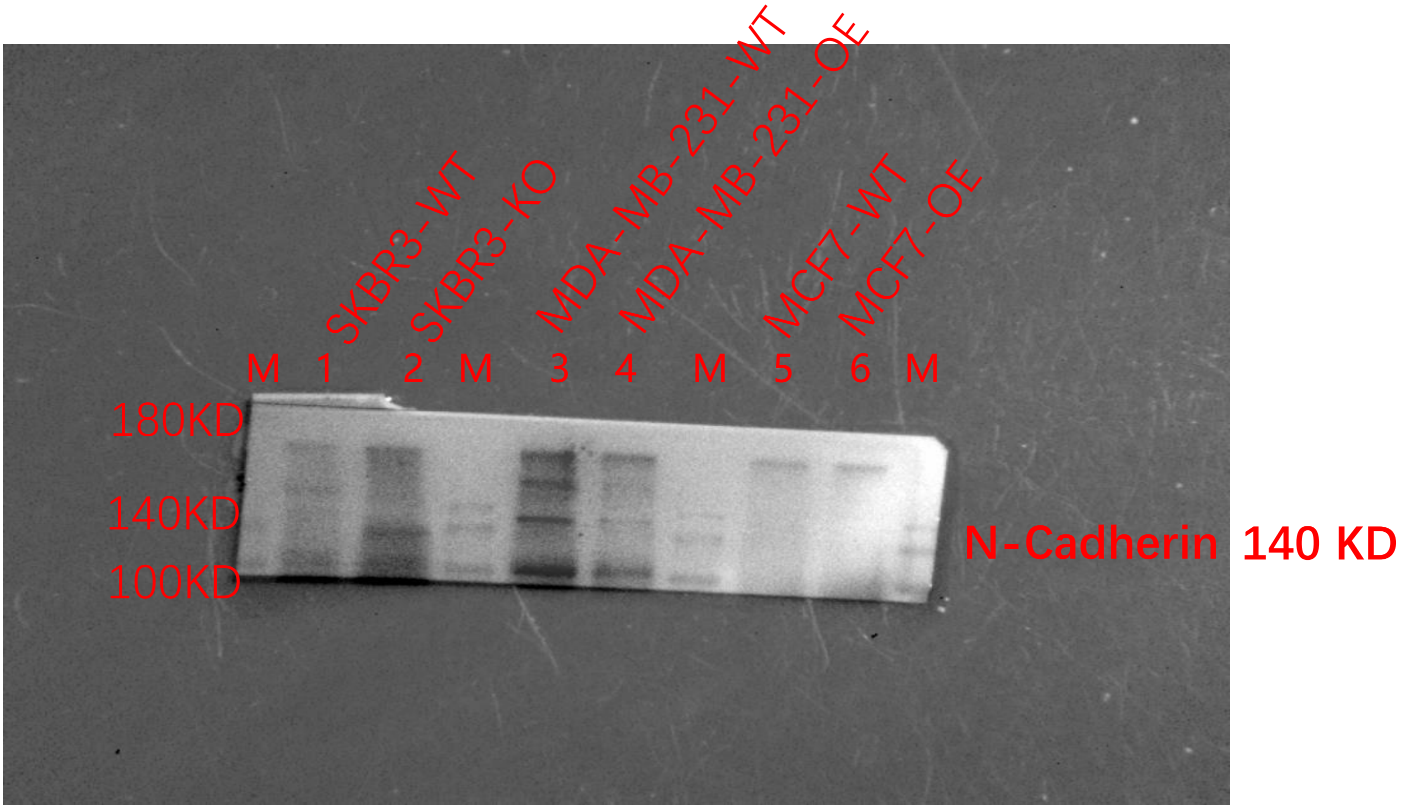

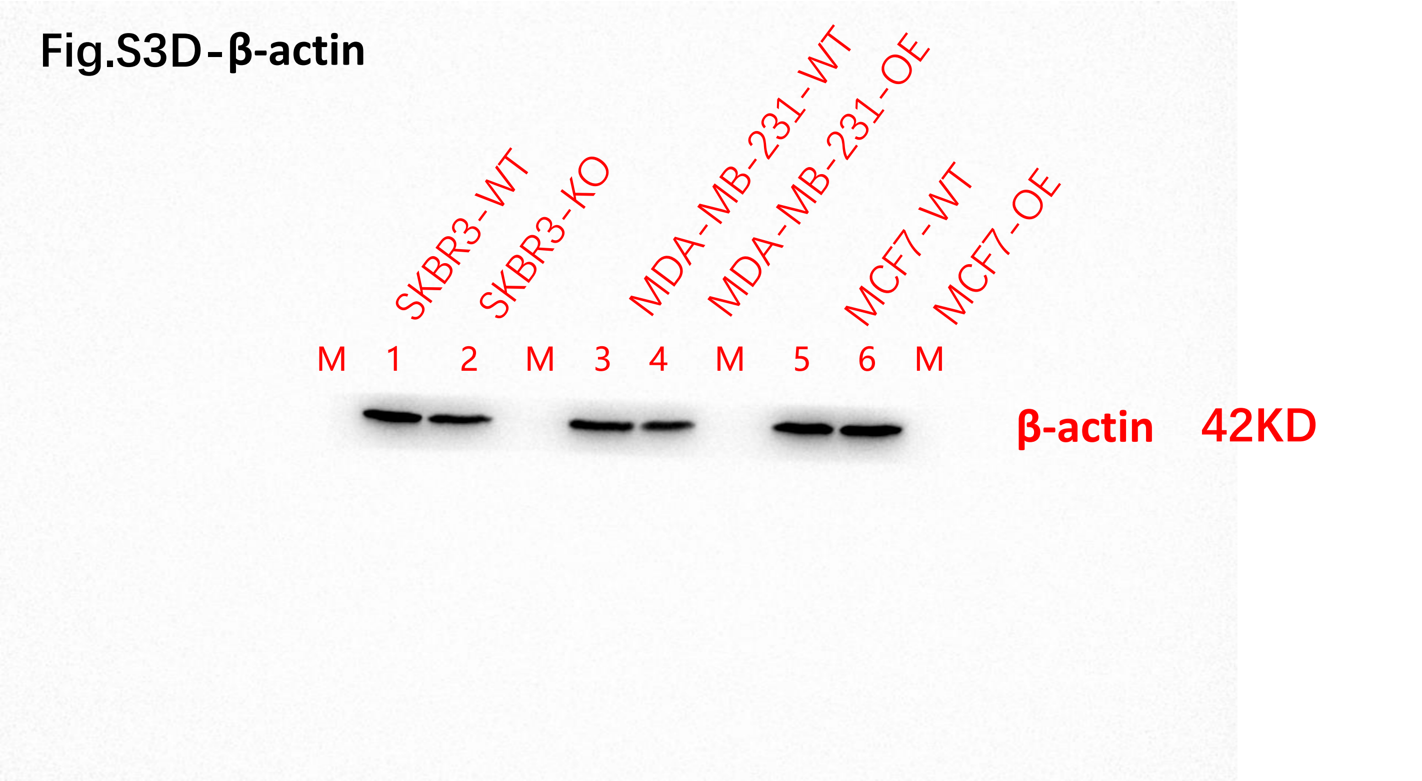

Fig.S3D-CD24

M:Marker

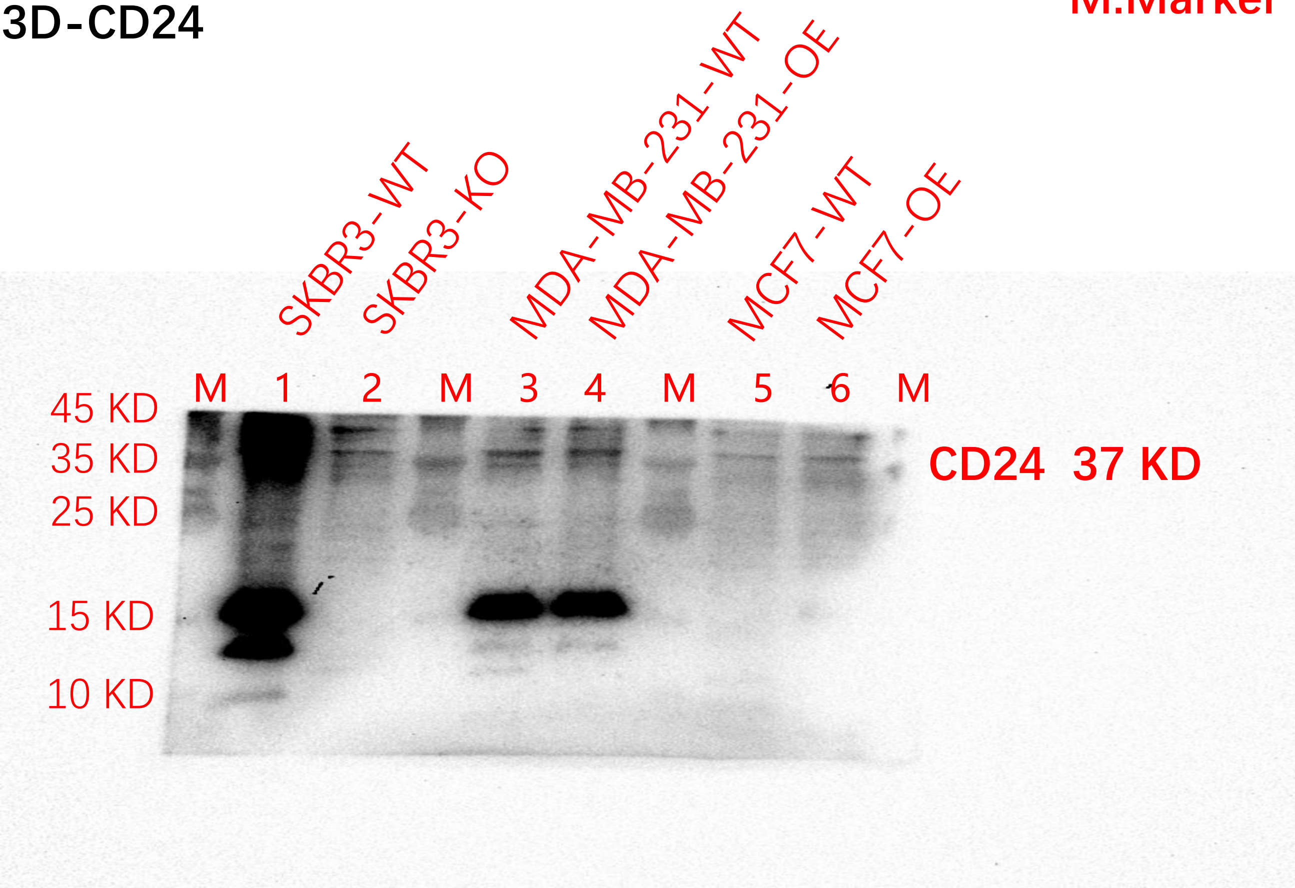

β-actin

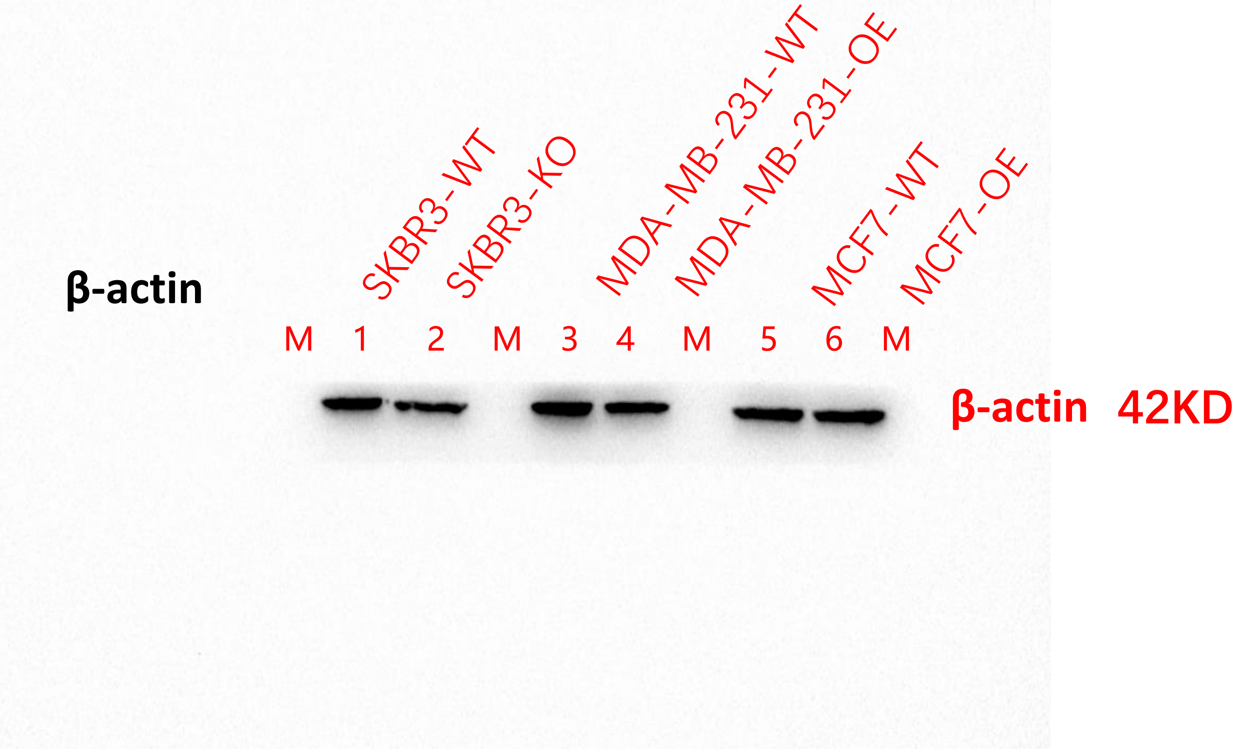

Fig.S3D-Vimentin

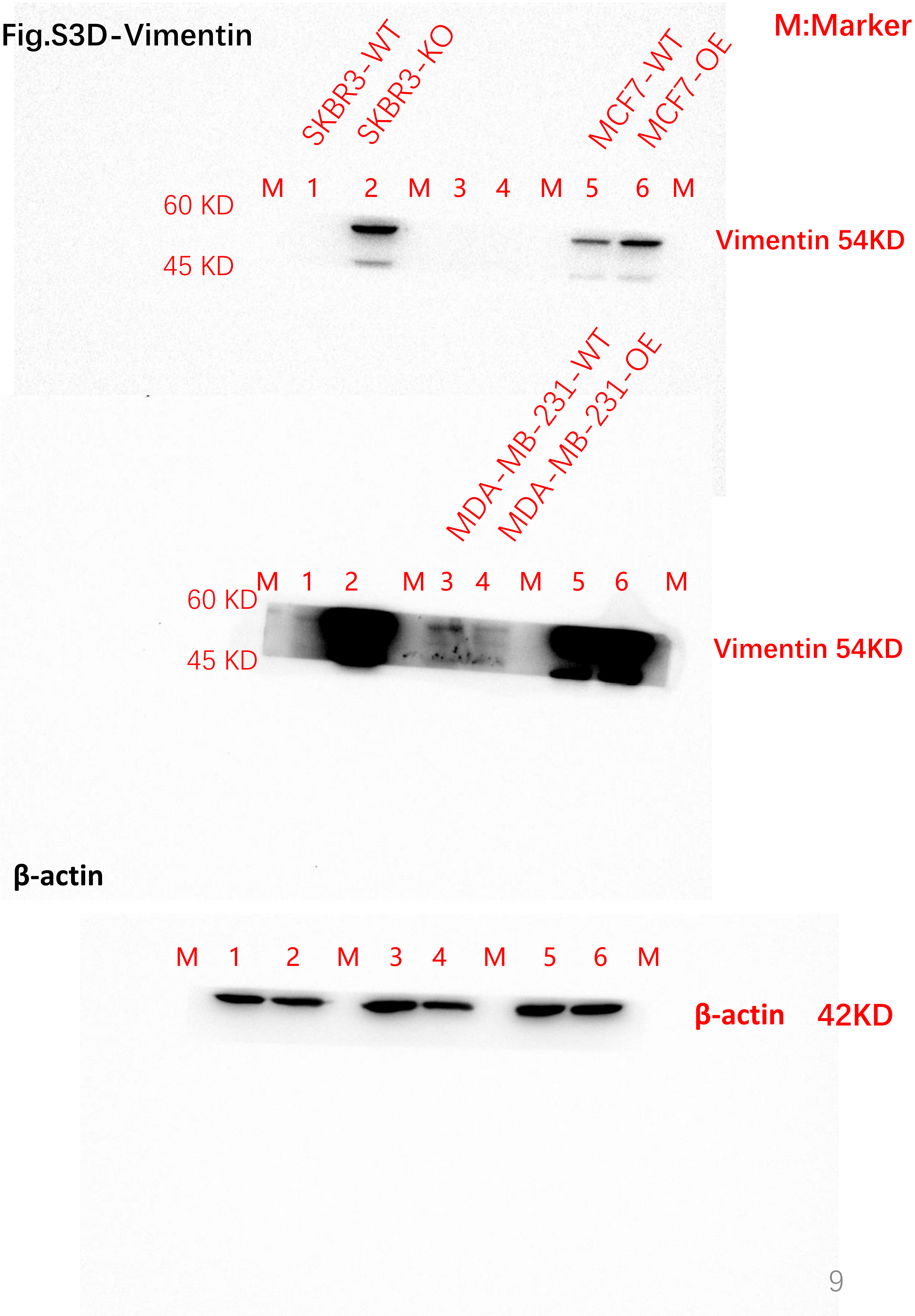

Fig.S3D-Twist1

M:Marker

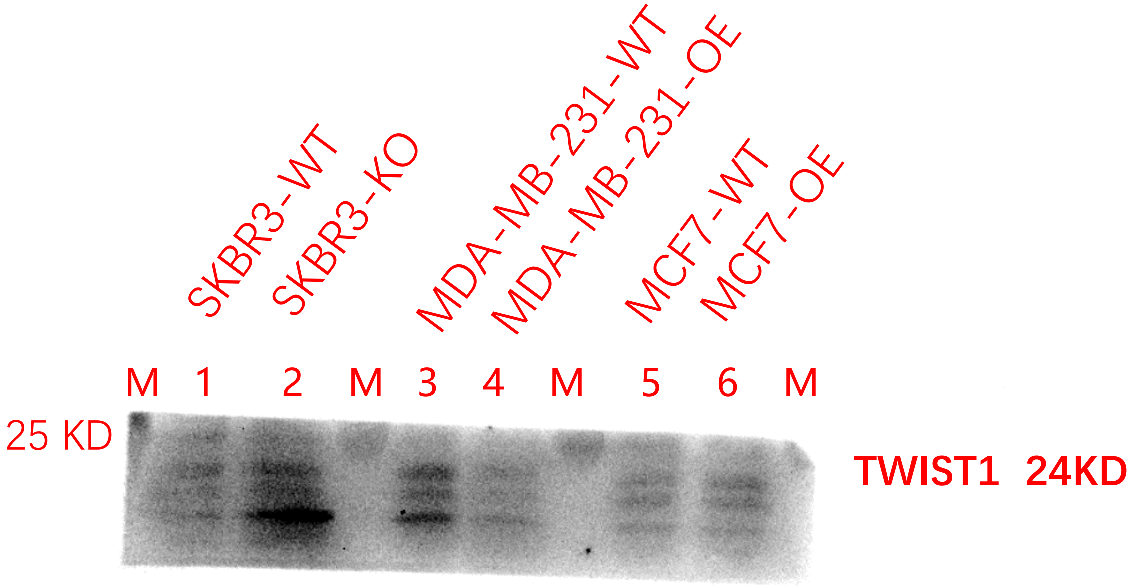

β-actin

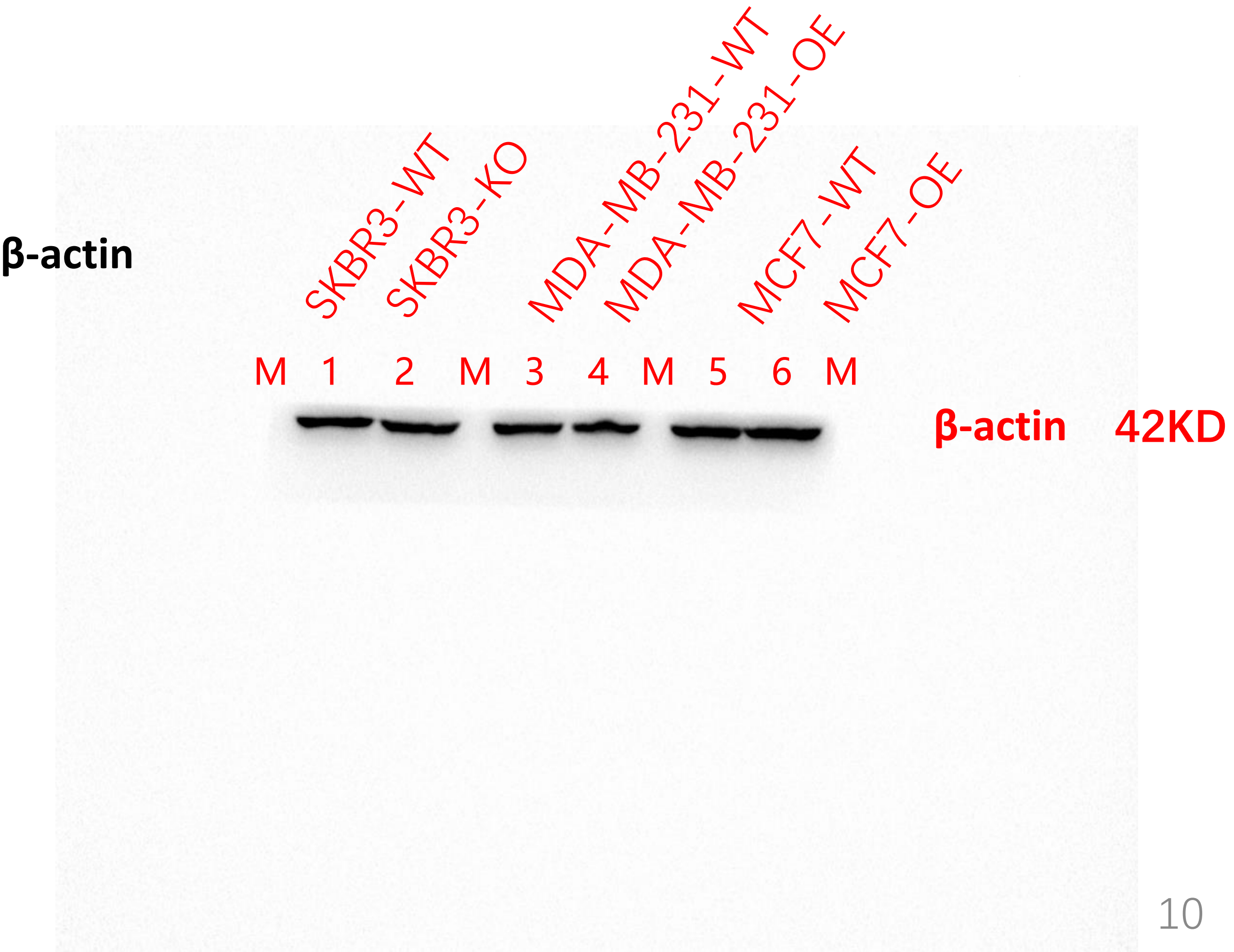

Fig.S4F-H3K27me3

M:Marker

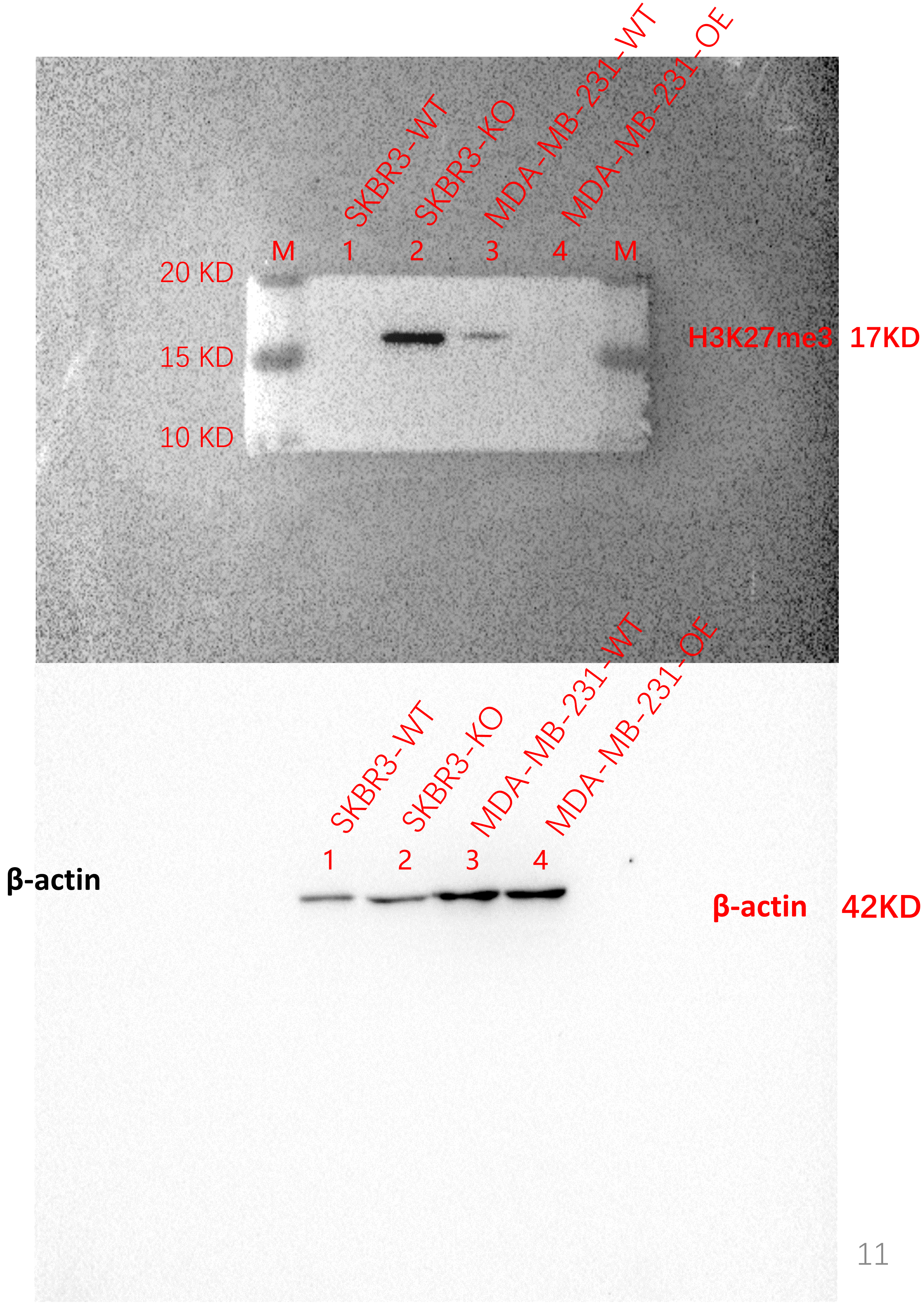

Supplement: Supplementary file 7 — Additional file 7: Full western blot image corresponding to Fig. 1H, Fig. S1G, Fig. 2B, Fig. S2D, Fig. S3D, and Fig. S4F. [file 13058_2023_1721_MOESM7_ESM.pdf]
